# Integrative Neuroscience of *Paramecium*, a “Swimming Neuron”

**DOI:** 10.1523/ENEURO.0018-21.2021

**Published:** 2021-06-04

**Authors:** Romain Brette

**Affiliations:** Sorbonne Université, Institut National de la Santé et de la Recherche Médicale, Centre National de la Recherche Scientifique, Institut de la Vision, Paris 75012, France

**Keywords:** ciliates, excitability, *Paramecium*, sensorimotor

## Abstract

*Paramecium* is a unicellular organism that swims in fresh water by beating thousands of cilia. When it is stimulated (mechanically, chemically, optically, thermally…), it often swims backward then turns and swims forward again. This “avoiding reaction” is triggered by a calcium-based action potential. For this reason, some authors have called *Paramecium* a “swimming neuron.” This review summarizes current knowledge about the physiological basis of behavior of *Paramecium*.

## Significance Statement

*Paramecium* is a unicellular organism that swims in fresh water by beating thousands of cilia. When it is stimulated (mechanically, chemically, optically, thermally…), it often swims backward then turns and swims forward again. This “avoiding reaction” is triggered by a calcium-based action potential. For this reason, some authors have called *Paramecium* a “swimming neuron.” This review summarizes current knowledge about the physiological basis of behavior of *Paramecium*.

## Introduction

Even the simplest behavior must engage at least a sensory organ, a large part of the nervous system, the body (muscles, skeleton), and the environment. Thus, understanding the biological basis of behavior requires an integrative approach, which remains highly challenging given the complexity of both the nervous and musculoskeletal systems of vertebrates. A fruitful research strategy is to study model organisms that are structurally simpler and have experimental advantages. For example, the biophysical basis of excitability was studied in the giant axon of the squid (Hodgkin, 1964), the molecular basis of learning and memory was studied in *Aplysia* (Kandel, 2009). A recent model organism to develop integrative approaches to behavior is *Caenorhabditis elegans*, with its 302 neurons and a known connectome (Schafer, 2018). In *C. Elegans*, modeling the entire organism and its interaction with the body and environment seems more feasible in principle (Cohen and Sanders, 2014; Cohen and Denham, 2019). Nevertheless, even in this more favorable situation, developing functional and empirically valid neuromechanical models of *C. elegans* remains very challenging. Two other recently introduced model organisms for this type of integrative work are *Hydra*, which has a few thousand neurons and the advantage of being transparent (Dupre and Yuste, 2017; Wang et al., 2020), and jellyfish *Aurelia aurita* (Pallasdies et al., 2019). Here, I will present a model organism that is significantly simpler as it consists of a single “neuron.”

**Figure 1.**
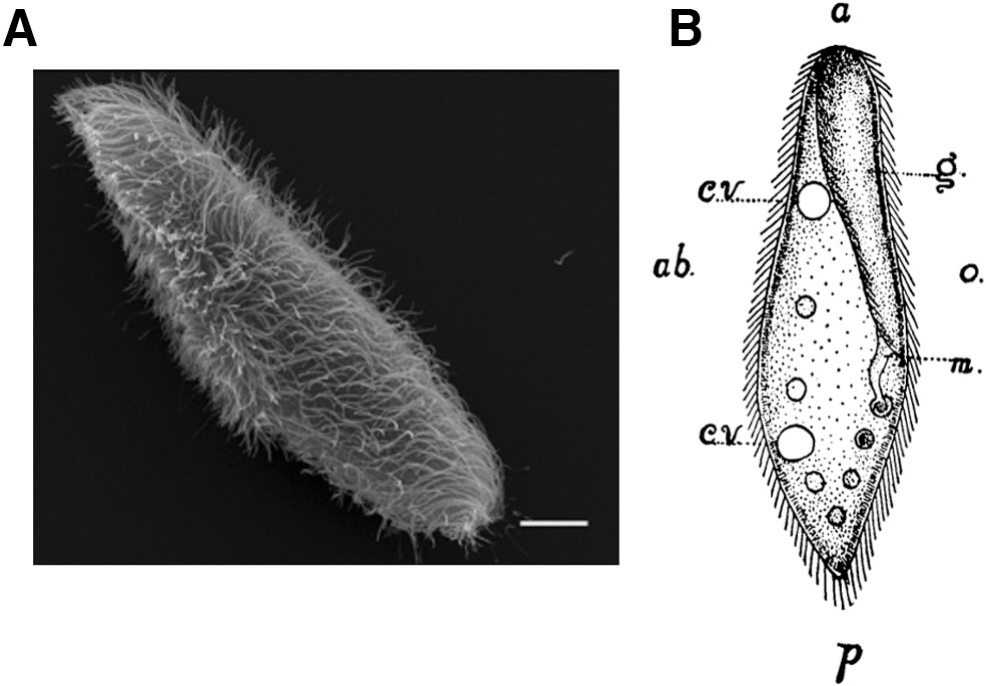
*Paramecium* morphology. ***A***, Scanning electron microscopy image of *P. tetraurelia*; scale bar: 10 μm (Valentine et al., 2012). ***B***, *Paramecium caudatum* (Jennings, 1899a), a large species (∼200 μm) with a pointed posterior end. a, anterior end; p, posterior end; g, oral groove; m, mouth; o, oral side; ab, aboral side; cv, contractile vacuole. The drawing also shows food vacuoles and cilia.

*Paramecium* is a single-cell eukaryote, 100–300 μm long depending on species (Fokin, 2010; Fig. 1), which has long been a model organism for many aspects of eukaryotic biology (Wichterman, 1986; Görtz, 1988). It is a ciliate that has been living in ponds and lakes all over the world for hundreds of millions of years (Parfrey et al., 2011), a fossil has been discovered in a 200 million-year-old piece of amber (Schönborn et al., 1999). Its abundance and large size made it a popular subject of behavioral study in the late 19th century; Jennings described his culture method as follows (Jennings, 1897): “A handful of hay or grass is placed in a jar and covered with hydrant water. In a few weeks the solution of decaying vegetable matter swarms with paramecia.”

**Figure 2.**
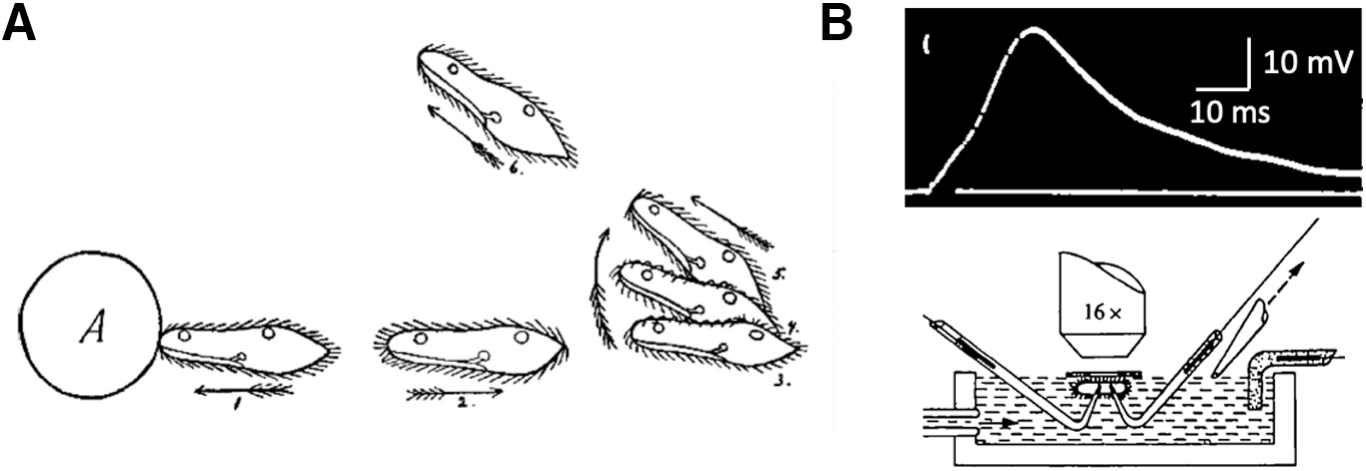
The avoiding reaction triggered by an action potential. ***A***, Avoiding reaction against an obstacle, as illustrated by Jennings (1906). ***B***, Action potential in response to a 2-ms current pulse (top), recorded with the hanging droplet method (bottom; from Naitoh et al., 1972 with permission).

*Paramecium* swims in fresh water by beating its thousands of cilia, and feeds on smaller microorganisms such as bacteria and algae. It is a prey for other microorganisms such as *Didinium*. As beautifully described by Jennings more than a century ago (1906), *Paramecium* lives in a rich sensory environment: it finds food by detecting and following chemicals produced by decaying plants and fellow paramecia; it moves toward the water surface by gravitaxis; it avoids obstacles thanks to its mechanosensitivity; it resists water currents by rheotaxis; it avoids bright light; it avoids hot and cold waters; it even communicates chemically. It typically swims in helicoidal paths interrupted by abrupt changes in direction called avoiding reactions, which form the “trial-and-error” basis of its behavior. When an unfavorable condition is met (obstacle, unwanted chemical), the avoiding reaction is triggered (Fig. 2*A*): *Paramecium* swims backward for a brief time, then turns and swims forward in a new direction. By this simple mechanism, *Paramecium* can navigate in crowded multisensory environments.

This avoiding reaction is triggered by an action potential produced by voltage-gated calcium channels located in the cilia (Fig. 2*B*; Eckert, 1972). These are L-type calcium channels related to the Ca_V_1 family found in neurons, heart and muscles of mammals (Lodh et al., 2016). A number of other ionic currents have been identified (Eckert and Brehm, 1979), and genes for many more ionic channels have been found in the genome, often homologs of mammalian channels (Martinac et al., 2008). Sensitivity to various sensory signals is provided by transduction into ionic currents, which may then trigger action potentials. Piezo channels, which convey mechanosensitivity in many species including mammals (Coste et al., 2010) have been identified in the genome. A rhodopsin-like protein has been identified in *Paramecium bursaria*, a photosensitive species (Nakaoka et al., 1991). In fact, many signaling pathways of neurons have been found in *Paramecium*, in particular calcium signaling pathways (Plattner and Verkhratsky, 2018), calcium release channels, pumps, calmodulin, centrin, calcineurin, SNARE proteins, cAMP and cGMP-dependent kinases, etc. For this reason, some authors have called *Paramecium* a “swimming neuron” (Kung and Saimi, 1985).

Many other motile unicellular organisms have rich behavior (Wan and Jékely, 2020) and produce action potentials, including microalgae (Eckert and Sibaoka, 1968; Harz and Hegemann, 1991; Taylor, 2009), other ciliates such as *Stentor* (Wood, 1991), other protists such as *Actinocoryne contractilis* (Febvre-Chevalier et al., 1986) and even bacteria (Kralj et al., 2011; Masi et al., 2015). One advantage of *Paramecium* is its large size, allowing relatively simple electrophysiological recordings (Naitoh and Eckert, 1972; Kulkarni et al., 2020). For this reason, there is a rich literature on *Paramecium* electrophysiology, mostly from the 1960–1980s (Eckert, 1972; Eckert and Brehm, 1979). In addition, *Paramecium* is still an active model organism in genetics, and benefits from many tools such as RNA interference (Galvani and Sperling, 2002); its genome has also been sequenced (Aury et al., 2006; McGrath et al., 2014).

I will first give an overview of the behavior of *Paramecium*, then I will explain how it moves with its body and cilia, and finally I will describe the physiological basis of behavior, with a special focus on the avoiding reaction. Most studies cited in this review were done on two species, *P. caudatum* and *P. aurelia*.

## The Life of *Paramecium*

### Swimming, feeding, reproducing

**Figure 3.**
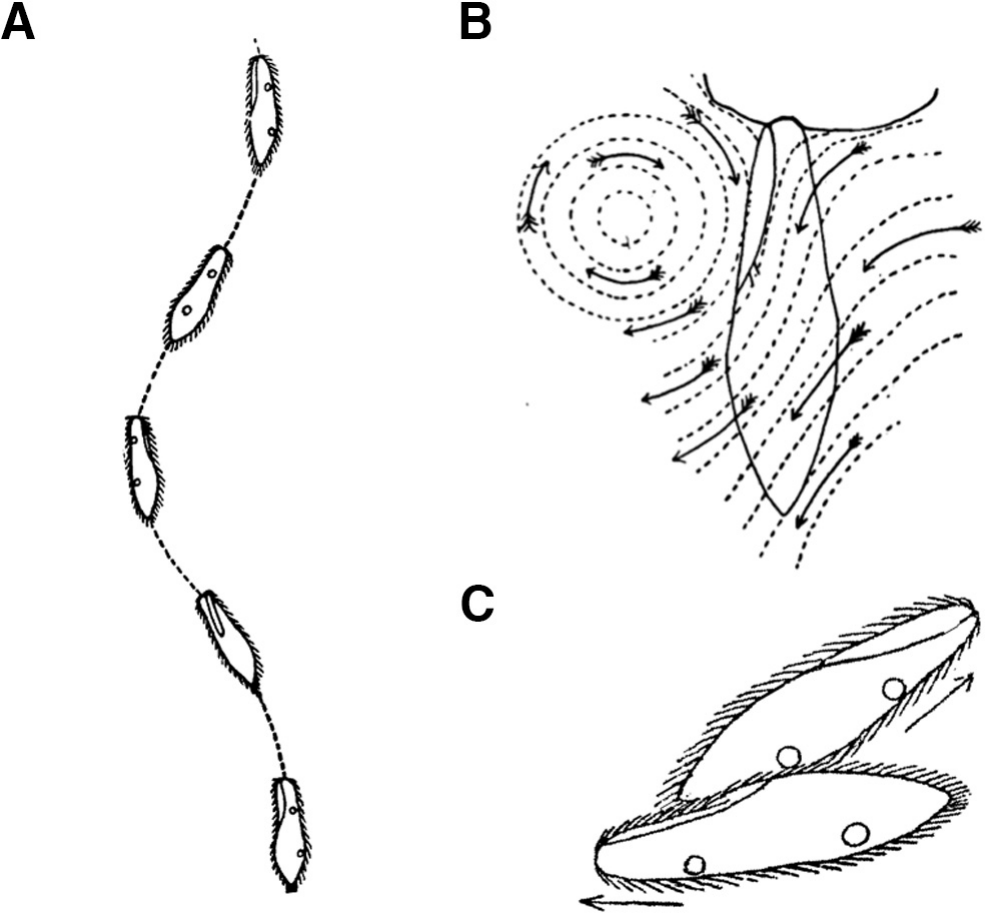
Swimming, feeding and reproducing. ***A***, Spiral swimming, with the oral groove facing the spiral axis (Jennings, 1899a). ***B***, Thigmotactic *Paramecium* resting against a fiber (Jennings, 1897). Arrows show water currents produced by oral cilia. ***C***, Two paramecia in conjugation (sexual reproduction; Jennings, 1904).

Behavior has been described in detail in articles and books by Jennings and a few contemporary scientists, in the late 19th and early 20th century (Jensen, 1893; Ludloff, 1895; Mendelssohn, 1895; Jennings, 1897, 1906); these observations would benefit from precise and systematic measurements with modern techniques. *Paramecium* lives in fresh water in various kinds of habitats, differing in temperature and composition. It swims in spiral paths at ∼1 mm/s by beating its thousands of cilia, revolving around its long axis at about one cycle per second, the oral groove facing the spiral axis (Fig. 3*A*; Jennings, 1899a; Bullington, 1930). These paths are occasionally interrupted by abrupt changes in direction, which can be preceded by a short period of backward swimming.

It is often found near the water surface, as it tends to swim against gravity (Jensen, 1893; p. 18). When it hits a solid surface such as glass or wood, it gives the avoiding reaction (Fig. 2*A*). But when it encounters some fibrous material such as a decaying plant or a piece of cloth, it may stall (Jennings, 1897). This behavior has been termed contact reaction or thigmotaxis (Fig. 3*B*). It can also occur on properly coated glass (Iwatsuki et al., 1996). The cilia in contact with the object are immobilized, and all the other cilia are quiet or quivering except the oral cilia, which beat strongly. In this situation, *Paramecium* may feed, for example on bacteria, yeast or algae. Food is brought into its oral groove by powerful cilia, which have different properties from locomotor cilia (Jung et al., 2014; Aubusson-Fleury et al., 2015).

A well-fed *Paramecium* can reproduce by fission every 6 h (Beisson et al., 2010a), depending on temperature (Krenek et al., 2011). Without food, *Paramecium* can survive for several weeks (Jackson and Berger, 1984). Starvation triggers sexual reproduction, where two individuals of opposite mating types attach to each other by the oral side and exchange genetic material (Fig. 3*C*). In *P. aurelia*, sexual reproduction can also occur by autogamy (with itself; Beisson et al., 2010b).

### Navigating

When *Paramecium* encounters a solid obstacle, it swims backward for a fraction of second, still revolving around its long axis, then the anterior end turns while the posterior end is still (Fig. 2*A*). This is called the avoiding reaction; it forms the basis of much of its behavior. According to Jennings, the organism always turns toward the same structurally defined side, the “aboral” side (away from the oral groove; Jennings, 1899a), although systematic measurements are lacking. But since it also revolves along its long axis, from a fixed viewpoint the change in direction may alternate between left and right. Thus, the change in direction may be considered as pseudo-random.

**Figure 4.**
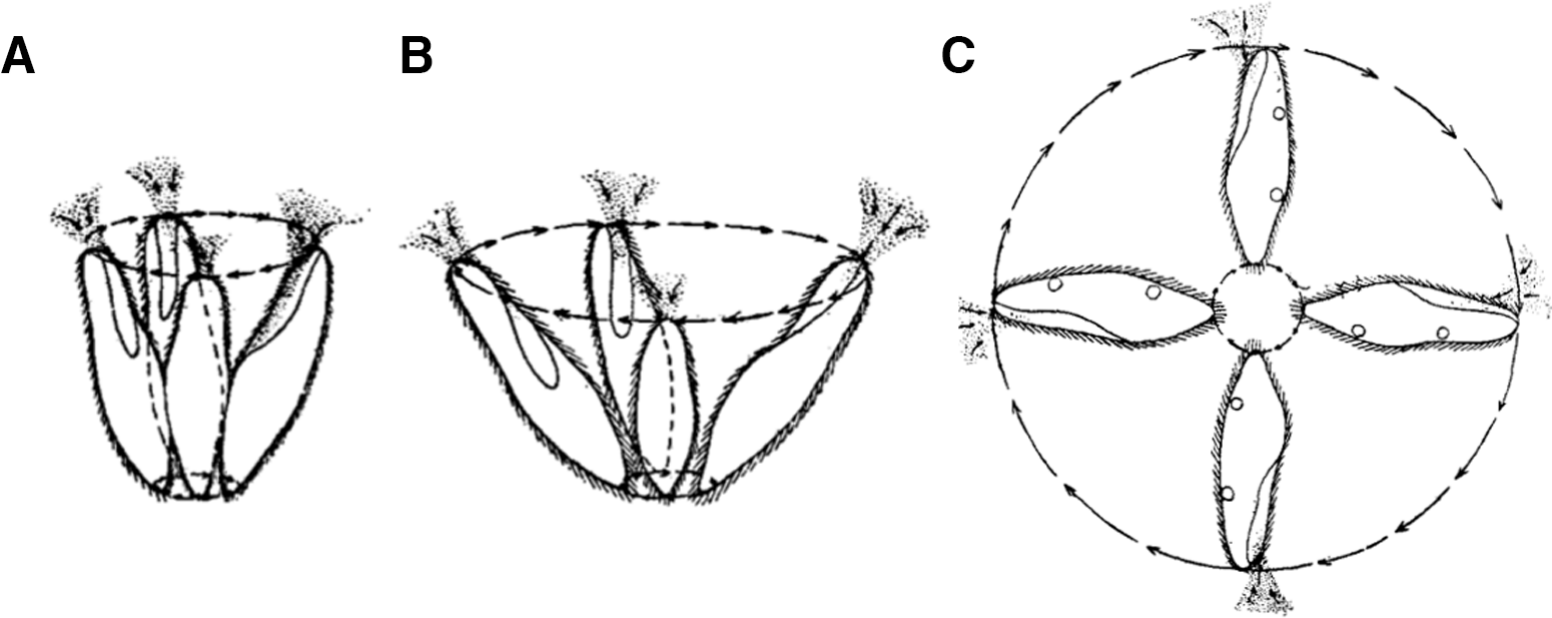
The avoiding reaction is graded (Jennings, 1904): swinging of the anterior end in a weak reaction (***A***), a strong reaction (***B***) and a very strong reaction (***C***).

The avoiding reaction is graded (Fig. 4). A weak stimulus may only trigger a gentle reorientation with no backward swimming (Fig. 4*A*), while a stronger stimulus induces backward swimming and reorientation (Fig. 4*B*). A very strong stimulus may trigger long backward swimming followed by turning a complete circle (Fig. 4*C*). This graded reaction parallels the graded action potential: the duration of backward swimming correlates with the stimulus-induced depolarization (Machemer and Eckert, 1973).

**Figure 5.**
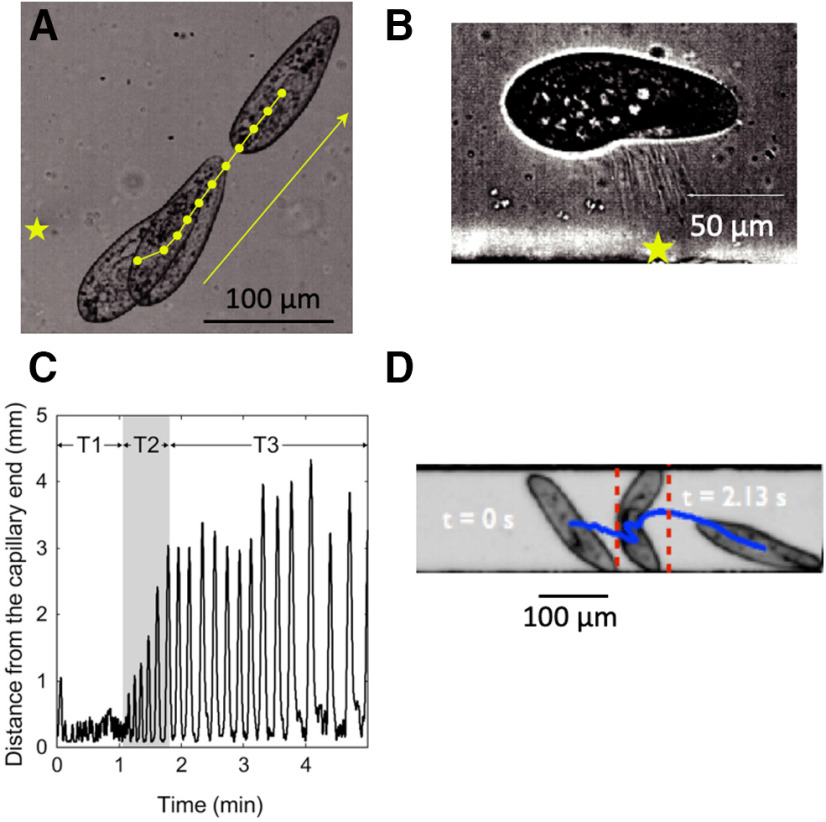
*Paramecium* navigation. ***A***, Escape reaction triggered by a heat stimulus (laser) near the posterior end (star; Hamel et al., 2011). ***B***, Sideways jumping from a strong heat stimulus (star) by throwing trichocysts (Hamel et al., 2011). ***C***, Trajectory of *Paramecium* in a 5-mm capillary, showing an increase in backward swimming after 1 min, corresponding to ∼40 avoiding reactions (Kunita et al., 2014). ***D***, Bending of *P. caudatum* in a 160-μm channel (Jana et al., 2015).

*Paramecium* also reacts when the rear is touched, but in a different way (Fig. 5*A*): it swims forward faster, by beating its cilia up to twice faster (Machemer, 1974). This speed increase is accompanied by a contraction along the longitudinal axis (Nakaoka and Machemer, 1990). This is called the escape reaction, first described by Roesle in 1903 (Roesle, 1903), then by Jennings (Jennings, 1904). Non-localized mechanical stimulation, as when shaking a tube of *Paramecium* culture, also induces an increase in swimming speed that can last for several minutes.

When stimulated by a strong heat using a laser (5–10°C increase), *Paramecium* can jump away from the stimulus (possibly sideways) within just 5 ms, at ∼10 mm/s (Hamel et al., 2011; Fig. 5*B*). To perform this feat, *Paramecium* throws trichocysts, which are sorts of needles docked near the membrane, thereby projecting itself in the opposite direction. The same behavior occurs in reaction to an appropriate chemical stimulus and in encounters with the predator *Dileptus* (Knoll et al., 1991).

When *Paramecium* swims in a narrow channel that does not allow it to turn, it may be trapped into a dead end, where it will give the avoiding reaction repeatedly, alternatively moving backward and forward against the wall (Kunita et al., 2014). But after a minute, the avoiding reaction suddenly becomes much longer (several millimeters), potentially allowing the organism to escape (Fig. 5*C*). When the channel is very narrow, *Paramecium* may also bend itself to move forward (Jana et al., 2015; Smith, 1908; Fig. 5*D*). The posterior end anchors onto the wall, presumably because tail cilia do not beat (Machemer and Machemer-Röhnisch, 1984; Ishikawa and Hota, 2006), while the anterior end slides along the other wall, causing the cell to bend until it can swim freely. Under some conditions, *Paramecium* can also slide along surfaces (Li and Ardekani, 2014; Nishigami et al., 2018; Ohmura et al., 2018). Some of this behavior is due not to physiological responses but to hydrodynamic interactions with surfaces (Berke et al., 2008; Lauga and Powers, 2009; Li and Ardekani, 2014; Ohmura et al., 2018).

Finally, in a water current, *Paramecium* orients itself with its anterior end directed up stream, a behavior called rheotaxis. According to Jennings (1906), rheotaxis derives from the avoiding reaction. When *Paramecium* swims along the water current, its cilia beat backwards and the water current opposes that movement. This acts as a mechanical stimulus which triggers the avoidance reaction. By trial and error, *Paramecium* turns until it faces the current. However, this remains an untested hypothesis. In a few other microorganisms, rheotaxis has been attributed to hydrodynamic effects (Bretherton and Rothschild, 1961; Marcos et al., 2012).

### Chemical sensing and social behavior

*Paramecium* is sensitive to a variety of chemical compounds (Jennings, 1899b; Nakatani, 1968; Dryl, 1973; Valentine et al., 2008). It is attracted by some substances, in particular bacterial metabolites (folate, acetate, glutamate, cAMP, biotin, ammonium, etc.), weak acids, carbon dioxide, colloidal solutions. These substances may indicate the distal presence of food, possibly components of the “phycosphere,” the rich interface between phytoplankton and bacteria (Seymour et al., 2017).

Other substances are repellent (e.g., alkaline solutions, quinine, ATP, GTP, GDP, NBT, Alcian Blue, Cibacron blue, Cytochrome c; Francis and Hennessey, 1995). Some of these molecules may signal the distal presence of a noxious condition. For example, Hennessey speculated that ATP and GTP are strong repellents because they are “blood-in-the-water signals” (Hennessey, 2005): these molecules are present at high concentrations in cells, and so their presence signals cell lysis, and whatever dangerous condition might have caused it.

Some substances only produce a reaction when *Paramecium* is subject to toxic doses (cane sugar, dextrose, urea), effectively killing it (Jennings, 1899b). For example, after some time, cane sugar induces plasmolysis, and then *Paramecium* begins to swim backward and forward repeatedly, possibly because of the induced depolarization. But many substances are toxic at doses much larger than the sensitivity threshold. In a number of cases, this sensitivity is conferred by specific membrane receptors, which can depolarize or hyperpolarize the cell (Van Houten, 1998) and possibly modulate ionic channels (Oami, 1996a,b).

**Figure 6.**
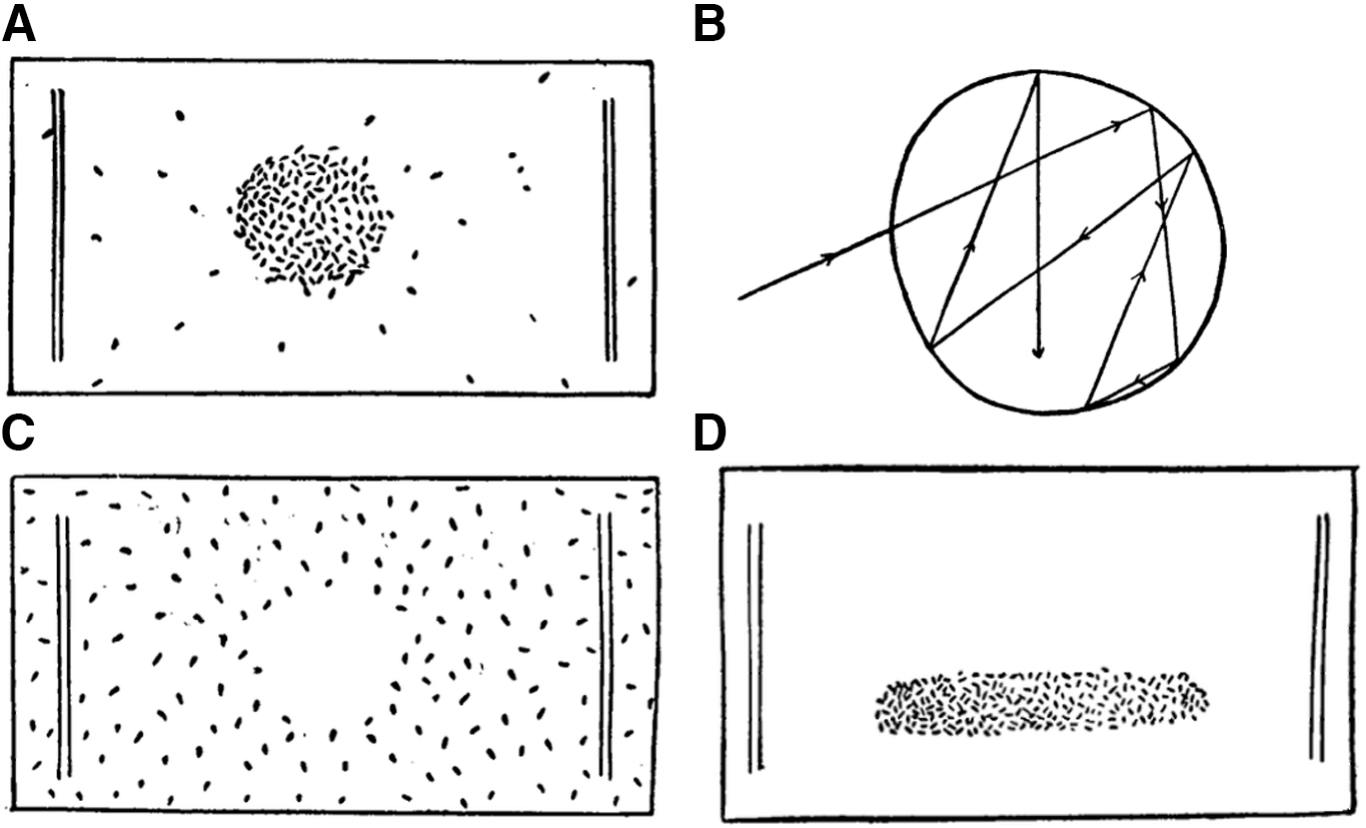
Chemotaxis and social behavior. ***A***, Gathering of paramecia in a drop of weakly acid solution (Jennings, 1899a). ***B***, Path followed by *Paramecium* in a drop of acid (Jennings, 1906). ***C***, paramecia avoiding a drop of sodium carbonate (Jennings, 1899a). ***D***, paramecia gathering in a cloud of carbon dioxide generated by their respiration (Jennings, 1899a).

In the 19th century, Jennings described the behavior of paramecia gathering in a drop of weak acid (Fig. 6*A*). He linked this behavior to the avoiding reaction. When *Paramecium* enters a drop of acid, its course is unchanged; but when it reaches the border of the drop, it gives the avoiding reaction and therefore remains in the drop (Fig. 6*B*). On the contrary, alkaline solutions are repellent: an avoiding reaction is triggered as soon as the alkaline solution is reached (Fig. 6*C*). More recently, various substances have been characterized as attractant or repellent based on the accumulation of paramecia in a test solution relative to a control solution, using different behavioral assays (Van Houten et al., 1975; Leick and Helle, 1983; Levandowsky et al., 1984; Nakazato and Naitoh, 1993; Valentine and Van Houten, 2016).

As previously mentioned, when stimulated, *Paramecium* turns to a structurally defined side (the aboral side, away from the mouth). Therefore, *Paramecium* is not attracted to a substance because it turns toward it. Rather, its behavior seems to result from trial and error: if attractant concentration increases, then *Paramecium* keeps on swimming in the same direction; if it decreases, then *Paramecium* changes direction. Jennings reported that the reaction of the organism is independent of where the chemical substance is applied; however, this may well depend on the compound because some chemoreceptors are spatially organized (Preston and Van Houten, 1987; Oami, 1996b).

For this reason, this behavior is sometimes named chemokinesis (changes in motility with chemical signals), which is more general than chemotaxis (movements toward a chemical stimulus; Houten, 1979). In particular, chemokinesis can result not only from modulation of the avoiding reaction (named klinokinesis), but also of swimming speed (named orthokinesis; Houten, 1978). Nonetheless, the chemical modulation of this apparently random motion can lead to motion toward the chemical source, and presumably to a preferred orientation of the body in the direction of the source (since the organism spends more time in the favored direction). There is some similarity with run-and-tumble chemotaxis in bacteria for which there is a dense literature (Berg, 2008; Sourjik and Wingreen, 2012), including theoretical (Berg and Purcell, 1977; Kollmann et al., 2005; Tu et al., 2008; Celani and Vergassola, 2010; Tu, 2013), and with pirouettes in *C. elegans* (Pierce-Shimomura et al., 1999).

A consequence of *Paramecium* attraction to weak acids is social behavior, as observed by Jennings (Jennings, 1897). By their respiration, *Paramecium* produces CO_2_, which is acid in solutions. At low concentration, *Paramecium* is attracted to CO_2_. It follows that paramecia tend to attract each other (Fig. 6*D*). This explains why gatherings can be observed at the bottom of a watch glass or at random positions in a tube. This may play an important role in feeding behavior, as it allows paramecia to collectively search for food.

Finally, *Paramecium* also has GABA_A_ and GABA_B_ receptors that can influence its behavior (Bucci et al., 2005; Ramoino et al., 2003, 2004). For example, the activation of GABA_B_ receptors inhibits the avoiding reaction. In addition, *Paramecium* releases GABA on stimulation. This release might act as a signal for other paramecia, or perhaps as an externalized spatial memory for exploration (as observed in slime mold; Reid et al., 2012), making the organism take a different action when it comes back to the same location. NMDA-like receptors have also been identified (Ramoino et al., 2014).

### The logic of *Paramecium* behavior

Many aspects of *Paramecium* behavior can be described as trial and error (1906). If its path is blocked by an obstacle, *Paramecium* withdraws then tries a new direction. If it encounters an undesirable chemical signal, it changes direction. If it leaves a desirable region, it withdraws and tries a new direction. This logic also applies to other sensory modalities. For example, when placed in a gradient of temperature, *Paramecium* accumulates in regions with temperature close to their culture temperature (Mendelssohn, 1895; Jennings, 1906). Again, this occurs by temperature-triggered avoiding reactions. When temperature changes away from culture temperature (whether this corresponds to a decrease or an increase), the avoiding reaction rate transiently increases; conversely, the avoiding reaction rate decreases when temperature gets closer to culture temperature (Nakaoka and Oosawa, 1977). This behavior is mediated by membrane potential changes (Tominaga and Naitoh, 1992) produced by cold-sensitive and heat-sensitive thermoreceptors (Tominaga and Naitoh, 1994; Kuriu et al., 1996, 1998).

*Paramecium* also shows photophobic responses to large changes in the intensity of visible light (mainly green, and red; Iwatsuki and Naitoh, 1982, 1983a,b; Hinrichsen and Peters, 2013). When *Paramecium* is kept in the dark and a bright light is turned on, it displays the avoiding reaction with a latency of around a second, then adapts over ∼15 s. As a result, *Paramecium* tends to accumulate in shaded regions. A related species, *P. bursaria*, is naturally highly sensitive to light and accumulates in lighted regions (Saji and Oosawa, 1974). This species harbors a symbiotic green alga named *Chlorella*: the alga provides photosynthetic products to its host while the host brings the alga in suitable light conditions. A moderate decrease in light intensity triggers an avoiding reaction, which makes *P. bursaria* seek light.

This trial-and-error behavior shares some similarity with the run-and-tumble behavior of bacteria (Berg, 1975). Macroscopically, trajectories of *Escherichia coli* resemble *Paramecium* trajectories, with helicoidal “runs” interrupted by “tumbles” where the cell changes direction randomly. Bacterial chemotaxis is enabled by concentration-dependent modulation of the tumbling rate: the tumbling rate decreases when concentration increases, while it is unchanged when concentration decreases. Thus, tumbling is not an avoiding reaction (it is not triggered by a concentration decrease). In *Paramecium*, the new direction is somewhat (pseudo-)random, but the turning event seems more deterministically related to environmental conditions than in bacteria. In other words, the avoiding reaction of *Paramecium* is more akin to a decision based on sensory inputs, than to a modulation of the spontaneous turning rate. This difference with bacteria may be because of a difference in scale: compared with bacteria, the membrane surface of *Paramecium* is at least two orders of magnitude larger, so that the signal-to-noise ratio is at least one order of magnitude larger; membrane potential fluctuations are ∼1–3 mV (Moolenaar et al., 1976; Nakaoka et al., 2009).

This simple logic of behavior calls for a couple of remarks in the context of neuroscience. First, it is somewhat surprising that a single spiking “neuron” can control relatively complex navigation in crowded multisensory environments, social behavior, and perhaps spatial memory. In terms of connectionism (Seung, 2012), *Paramecium* is a zero-connectome organism, and yet it can accomplish a variety of ecologically relevant tasks. This arises not from the complexity of the cell, which is electrically much simpler than a single pyramidal cortical neuron (it is isopotential), but rather from the interaction between this spiking cell and the environment, together with the exploratory properties conferred by the pseudo-random nature of the effect of a spike. This highlights the importance of embodiment and coupling with the environment, which are increasingly appreciated in cognitive science and philosophy of mind (Maturana and Varela, 1973; Powers, 1973; Gibson, 1979; Brooks, 1991; Bickhard and Terveen, 1996; Hurley, 2001; O’Regan and Noë, 2001; Ahissar and Assa, 2016; Pezzulo and Cisek, 2016; Brette, 2019).

Second, “control” may not be the right term to describe the relation between spiking and behavior. Motor control is classically described as feedforward or feedback (Wolpert and Ghahramani, 2000). In feedforward control, internal models are used to plan movements, and specific sets of neurons are recruited to trigger the appropriate movements. In *Paramecium*, spiking produces a single type of movement, regardless of the goal or stimulus: it does not move by planning specific movements. In feedback control, actions are taken that reduce the difference between the observed state and a desired state. In *Paramecium*, an action is also taken when the observed state is undesirable, but that action is not directed toward the goal, rather, it is (pseudo-)random. Thus, *Paramecium* movements are based neither on feedforward nor on feedback control, but rather on exploration and selection (trial and error). This is reminiscent of the Darwinian insight that an apparently goal-directed process can occur through random exploration and elimination of unsuccessful choices, rather than by either planning or steering.

### Adaptation

**Figure 7.**
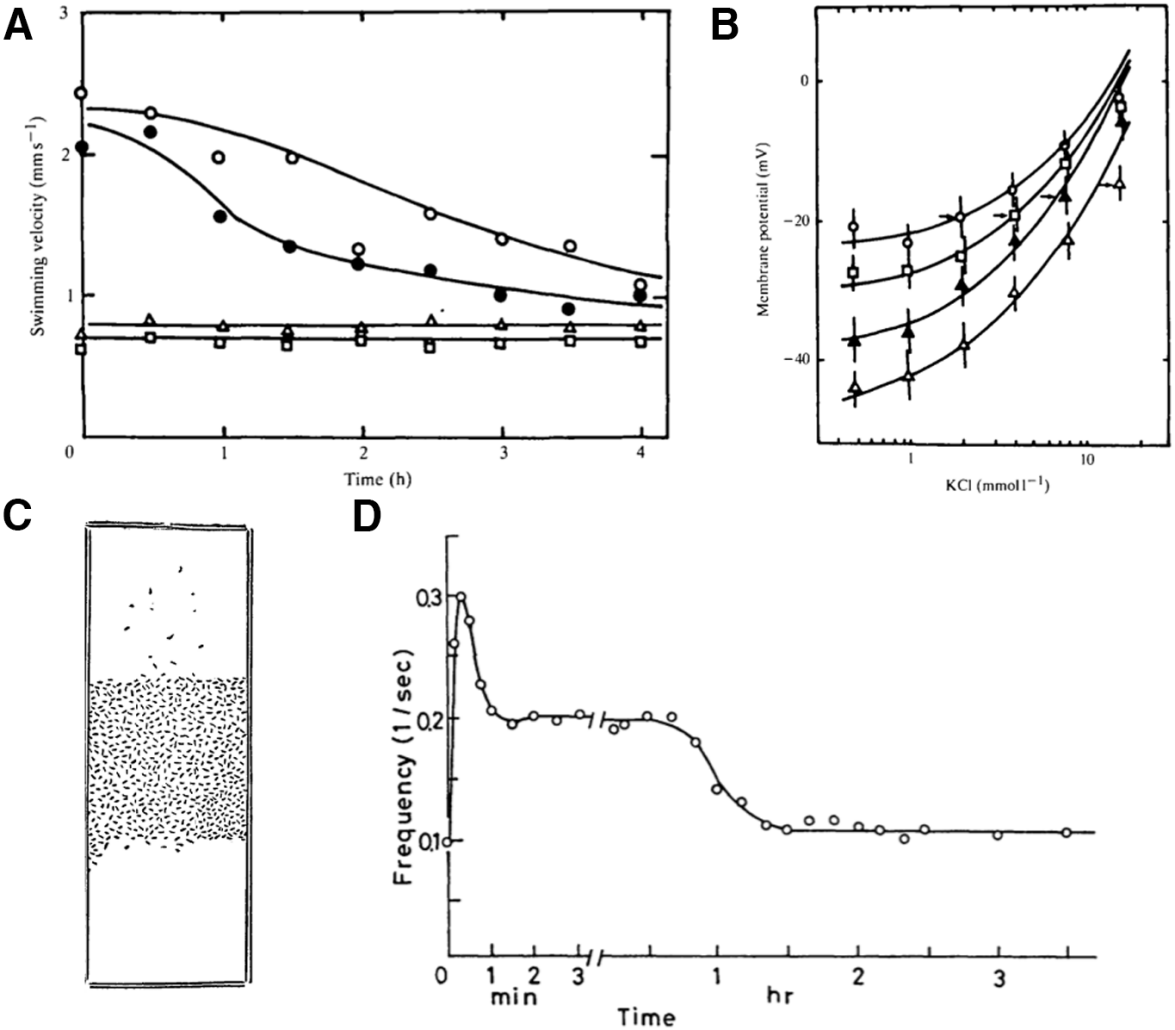
Adaptation. ***A***, Change in swimming velocity when *Paramecium* adapted to a solution of 0.25 mm CaCl_2_ and 4 mm KCl is transferred to a solution of 0.25 mm CaCl_2_ and 1 mm (open circles), 2 mm (closed circles), 4 mm (squares), or 16 mm (triangles) KCl (from Oka et al., 1986). ***B***, Resting potential versus KCl concentration for cells adapted to 2 mm, 4 mm, 8 mm, and 16 mm KCl (top to bottom; Oka et al., 1986, with permission). Arrows indicate the adapted state. ***C***, Accumulation of *Paramecium* in a warm region (Jennings, 1899a). The top of the slide is placed on a 40°C bath while the bottom rests on ice. ***D***, Change in avoiding reaction rate after paramecia cultured at 25°C are transferred to 30°C (from Nakaoka et al., 1982, with permission). Note the change in time scale.

*Paramecium* lives in habitats of diverse ionic composition. Changes in ionic composition directly affect ionic currents and reversal potentials, and therefore can potentially interfere with behavior. For example, moderate changes in cation concentration can alter swimming velocity (Machemer, 1989; Nakaoka et al., 1983). More critically, an increase in potassium concentration can inhibit the avoiding reaction through depolarization-induced inactivation of the ciliary calcium channels (Oka and Nakaoka, 1989), making the organism unresponsive to stimulation. Remarkably, after a couple of hours, behavior returns to its normal state before the medium changed (Oka et al., 1986; Fig. 7*A*). In parallel, the resting potential changes after a medium change then decays back to its original value (Fig. 7*B*). This homeostatic regulation appears to be mediated by changes in channel permeability. With a more prolonged (48 h) exposure to a high potassium solution, more complex changes in excitability can occur, with enhanced responses to Mg^2+^ and Na^+^ (Preston and Hammond, 1998).

Temperature also affects ionic channel properties and the entire metabolism of the organism, as well as hydrodynamic properties (viscosity of water). For example, when temperature is lowered, the ciliary calcium current is smaller and slower, action potentials are smaller and broader, cilia reverse with longer latency and for a longer time (Machemer, 1974). As previously discussed, *Paramecium* has a thermoregulation mechanism based on movement: using the avoiding reaction, it navigates toward waters of a preferred temperature (Fig. 7*C*). However, this mechanism is insufficient if the medium changes temperature globally. Remarkably, in this case, *Paramecium* adapts over a couple of hours: behavior returns to normal and the new temperature becomes the preferred temperature (Nakaoka et al., 1982; Fig. 7*D*). This behavioral adaptation correlates with changes in electrophysiological properties, in particular of the ciliary calcium conductance (Martinac and Machemer, 1984).

### Learning

Beyond adaptation, there is an important literature on learning in *Paramecium* and other ciliates. Unfortunately, as reviewed by Applewhite (1979), many of those studies are difficult to interpret as they lack appropriate controls or observations. In a series of papers (Gelber, 1952, 1956, 1957, 1958, 1962a,b), Gelber showed an apparent reinforcement of behavior with a food reward (see (Gershman et al., 2021) for a recent commentary). A platinum wire is lowered repeatedly into a depression slide with paramecia. If the wire is intermittently baited with bacteria, then more and more paramecia cling to the wire, even when a clean wire is finally lowered into the slide. What might be the stimulus? Gelber (1956) noted that the behavior was not observed when paramecia were tested in the dark, suggesting that perhaps paramecia, with permission developed an attraction to a reflection or shadow cast by the wire.

These observations were controversial, because it was objected that lowering the baited wire introduces bacteria in the fluid, to which paramecia are then attracted even when the wire is removed or cleaned (Jensen, 1957). In support of this interpretation, Katz and Deterline (1958) replicated Gelber’s main findings but found that stirring before the final test destroyed the observed behavior. Naturally, this could be interpreted as an erasure of learning because of the mechanical disturbance, but perhaps more crucially, they found that Gelber’s observations could be reproduced when the entire experiment (not just the test) was done in the dark, effectively removing any distal sensory stimulus by which paramecia may be able to recognize the wire. A plausible explanation, in line with informal observations reported in this set of studies, is that feeding reduces the activity of paramecia so that they tend to stay near the wire, and promotes thigmotaxis so that they tend to adhere more easily to the wire. In this case, the procedure would indeed reinforce a behavior, namely the feeding behavior, but not a stimulus-specific behavior. More detailed observations seem necessary to understand the phenomenon.

Another phenomenon that has attracted some attention is tube escape learning, first described by French in 1940 (French, 1940). A single *Paramecium* is placed in a drop and a thin tube is lowered into it. The organism is drawn into the tube by capillarity. It then escapes from the bottom after ∼30 s. When the experiment is repeated, escape time decreases to around 15 s after a few trials. French states that after the initial trials, paramecia go and back and forth in the tube only a few times then take “one long dive to the bottom.” The faster escape persists for at least 2 h (Huber et al., 1974), which seems to rule out the possibility that *Paramecium* simply adapts to the mechanical stimulus of capillary suction. This phenomenon has been robustly reproduced by several authors (Hanzel and Rucker, 1972; Applewhite and Gardner, 1973), but its basis is unclear. Applewhite and Gardner (1973) proposed that *Paramecium* released some substance in the tube that then influences future behavior, but this hypothesis contradicts earlier results by Hanzel and Rucker (1972) showing the same performance improvement in multiple paramecia with the same tube. Studies of tube escape learning in *Stentor*, another ciliate, suggest that the phenomenon is related to gravitaxis (Bennett and Francis, 1972; Hinkle and Wood, 1994). Performance improvement is seen only when the tube is vertical, not when it is horizontal, where escape is fast from the first trial. This suggests the following (speculative) explanation: in a vertical tube, paramecia are trapped near the top because of negative gravitaxis, then prolonged confinement (perhaps signaled by frequent avoiding reactions) inhibits the normal gravitactic behavior, so that the organism can escape to the bottom.

Finally, Hennessey et al. (1979) managed to train *Paramecium* to react to sounds. When a tone is played by a speaker below the slide, *Paramecium* shows no reaction. However, when the tone is paired with electrical stimulation triggered in the middle of the tone, *Paramecium* reacts to the stimulus with an avoiding reaction, then after a few trials gives an avoiding reaction at the onset of the tone, in anticipation of the electrical stimulus. The authors demonstrate extinction (reaction disappears when sound is presented alone), retention and specificity (reacting specifically to a 300-Hz tone or to a 500-Hz tone). The physiological basis is not known.

Armus and colleagues (Armus et al., 2006a,b; Mingee and Armus, 2009) trained paramecia to go to a lighted region. The bath is split into two compartments, one in the dark, the other one in light. Initially, *Paramecium* spends more time in the dark compartment, because of photophobia. Training consists in electrically stimulating the cell when it enters the compartment of the cathode. After training, *Paramecium* spends more time than before in the cathodal half, which now only differs by its lighting. However, if stimulation is triggered in the anodal half, then after training *Paramecium* spends less time in that half. Therefore, the phenomenon does not seem to be based on an association between the electrical stimulus and the light stimulus. A plausible interpretation is the following. As is known from studies of galvanotaxis (Ludloff, 1895; Dale, 1901), electrical stimulation makes *Paramecium* move toward the cathode. Stimulation in the lighted cathodal compartment then makes *Paramecium* spend more time in light, which results in adaptation of the photophobic behavior. Thus, after training, *Paramecium* spends more time than before in the lighted compartment. This interpretation is supported by the observation that the “trained” behavior only occurs when the cathodal compartment is lighted during training (Alipour et al., 2018), and by the absence of retention (Mingee, 2013).

In summary, although the existing literature is complex, there is clear evidence of behavioral plasticity in *Paramecium*. Some can be categorized as adaptation, and there is at least one documented case of learning (Hennessey et al., 1979), understood as a persistent stimulus-specific change in behavior.

## The Motor System of *Paramecium*

### How *Paramecium* swims

**Figure 8.**
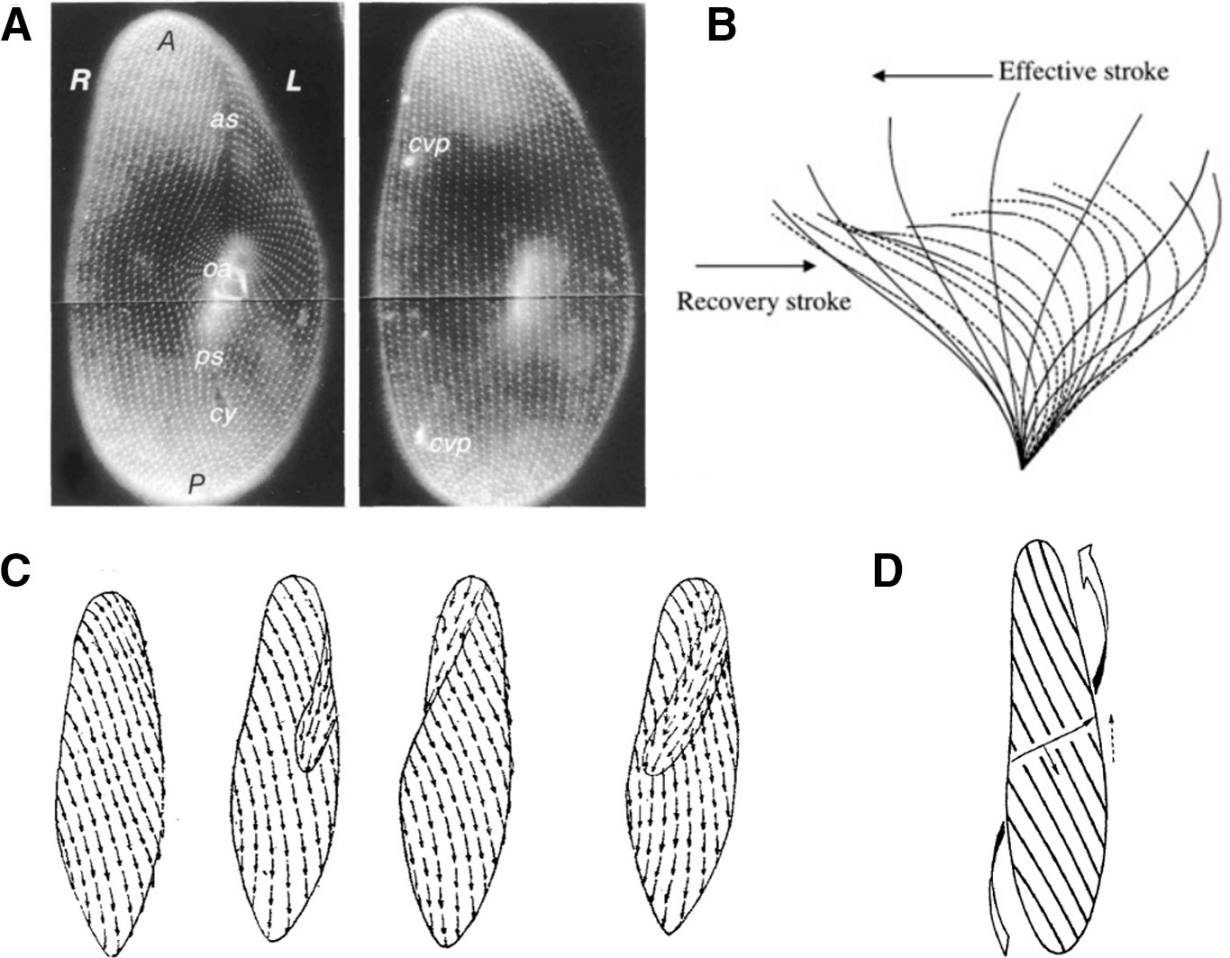
Spiral swimming. ***A***, Organization of ciliary basal bodies on the oral (ventral) and aboral (dorsal) side (from Iftode et al., 1989, with permission). ***B***, Ciliary beat cycle: power stroke (or effective stroke) and recovery stroke (Omori et al., 2020). ***C***, Water currents produced by cilia for different orientations of *Paramecium* (Jennings, 1904). In the oral groove, currents are oriented toward the mouth. ***D***, Metachronal waves represented by parallel lines, progressing transversally, with cilia’s power stroke oriented toward the right and rear (from Machemer, 1972, with permission). Cilia on parallel lines are at the same phase of the beat cycle. The curved arrow shows the direction of movement.

In the absence of any stimulus, *Paramecium* swims in spirals. *Paramecium* is covered by several thousand cilia (Fig. 8*A*; ∼4000 cilia in *Paramecium tetraurelia*; Aubusson-Fleury et al., 2015; for precise counts and spatial pattern, see Iftode et al., 1989), each ∼10 μm long and 0.2 μm thick (Eckert and Naitoh, 1970), similar to other motile cilia of eukaryotes, including mammals (Ishikawa, 2017). In forward swimming, each cilium beats at 10–20 Hz (Fig. 8*B*), with a power stroke toward the right and rear on the visible surface (Fig. 8*C*). Thus, on the hidden surface (further from the observer), cilia beat toward the left and rear. This results in a forward movement with a rotation around the longitudinal axis, as in unscrewing (over to the left; Fig. 8*D*). The typical velocity is ∼1 mm/s (Machemer et al., 1991).

The spiral is wider than the cell’s width, as first described by Jennings (1901) and later by Bullington (1930). A possible reason is that cilia in the oral groove beat in a specific direction, toward the mouth, which counters the movement produced by the other cilia. A recent study has shown indeed that properties of oral cilia differ from other cilia (Jung et al., 2014). This may explain why the trajectory describes a wide spiral, with the oral side always facing its axis (Fig. 3*A*; Párducz, 1967).

Properties of spiral swimming can vary, in particular its speed and width. *Paramecium* can also swim backward, with an effective stroke toward the front and slightly to the right. Thus, in backward swimming, the movement is not the symmetrical of forward swimming: the cell still rotates in the same direction.

Cilia beating is coordinated over the cell in the form of *metachronal waves*, which progress over the surface at ∼1 μm/ms (Párducz, 1967; Fig. 8*D*). These waves encircle the body in spirals (Párducz, 1967; Machemer, 1969). Cilia beat against the direction of the wave, but not at 180°, a pattern called “dexio-antiplectic.” This particular kind of motor coordination is functionally important. A key characteristic of swimming microorganisms is they live at low Reynolds number (R ≈ 0.1 for *Paramecium*; Purcell, 1977), that is, inertial forces are small compared with viscous forces (as if a human were trying to swim in honey). As a consequence, the swimmer stops as soon as cilia stop beating. Therefore, if cilia beating were synchronized over the entire body, then the swimmer would move forward in regular discontinuous steps. In fact, this can happen in the escape reaction: a strong heat stimulus near the posterior end induces a synchronous power stroke of the cilia (as in the butterfly stroke; Hamel et al., 2011), which results in a transient speed increase immediately followed by an almost complete stop, before the metachronal pattern is reestablished. If on the contrary cilia beating were completely disorganized (which can happen transiently in the avoiding reaction), then neighboring cilia might beat in inconsistent directions and this is not an efficient way of swimming. In fact, it has been shown that the metachronal pattern optimizes the energetic efficiency of swimming (Gueron and Levit-Gurevich, 1999; Osterman and Vilfan, 2011).

It was once postulated that ciliary coordination might be electrically controlled by the cell, but *Paramecium* is essentially isopotential (Eckert and Naitoh, 1970). Instead, cilia coordination is mediated by hydrodynamic interactions (Machemer, 1972; Guirao and Joanny, 2007) and mechanical coupling through the compliant body (Narematsu et al., 2015), in the absence of any central agency. This illustrates the concept of embodiment in motor neuroscience: part of the problem of efficient coordination is solved not by manipulating body representations, but by direct physical interaction of the body with its immediate environment (Tytell et al., 2011). In the case of microorganisms such as *Paramecium*, the results of this physical interaction can be understood precisely, thanks to an abundant literature on the mechanics of cilia and flagella (Blake and Sleigh, 1974; Sartori et al., 2016; Wan, 2018) including mathematical models (Dillon et al., 2007; Yang et al., 2008), as well as on the hydrodynamics of swimming microorganisms (Keller and Wu, 1977; Lauga and Powers, 2009; Jung et al., 2014).

### How *Paramecium* moves upward

**Figure 9.**
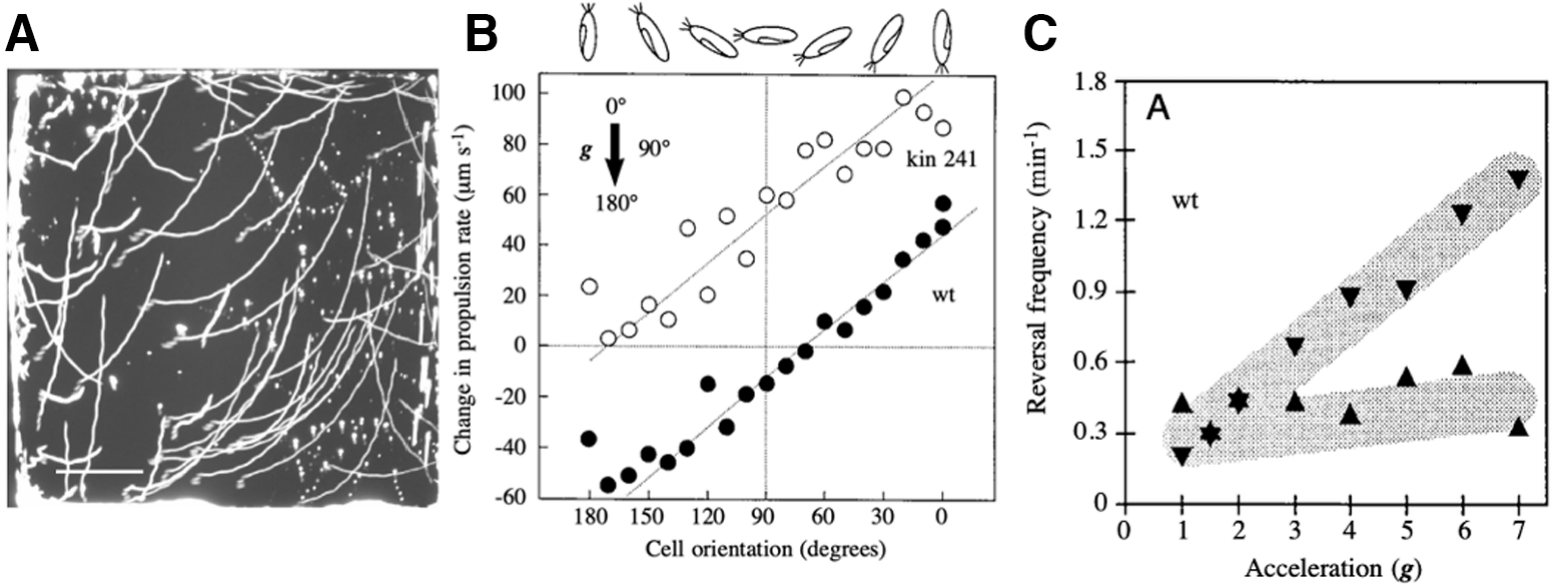
Gravitactic behavior of *Paramecium*. ***A***, Upwardly curved trajectories of *Paramecium* in a vertical chamber (from Roberts, 2010, with permission). ***B***, Velocity change (corrected for sedimentation) as a function of cell orientation (from Nagel and Machemer, 2000, with permission), open circles correspond to a morphologic mutant. ***C***, Avoiding reaction frequency as a function of acceleration in a centrifuge microscope, after 4 h of equilibration (from Nagel and Machemer, 2000, with permission). Triangles indicate cell direction.

As many other microorganisms (Häder and Hemmersbach, 2018), *Paramecium* tends to aggregate near the water surface, despite the fact that it is slightly heavier than water (∼4%), a puzzling phenomenon which has attracted an abundant literature, first described in detail by Jensen in 1893 (Jensen, 1893). When observed in a vertical plane, trajectories are curved upward (Roberts, 2010; Fig. 9*A*). The earliest explanation, the gravity-buoyancy model, postulates a mismatch between the buoyancy center and the gravity center (Verworn, 1889): this could generate a torque making the body align with gravity. Roberts (Roberts, 1970, 2010) argued that density inhomogeneities are unlikely to be sufficient to account for the observations, and instead proposed a drag-gravity model: as the posterior end is larger than the anterior end, the viscous drag differs and the posterior end falls more rapidly than the anterior end; thus, the cell turns upward. However, Jensen (1893) and later Kuznicki (1968) observed that dead or immobilized cells fall with no preferred orientation, although this is questioned by Roberts (Roberts, 1970). This would discard both passive orientation mechanisms. The propulsion-gravity model (Winet and Jahn, 1974) is a more complex proposition, which links gravitaxis with ciliary beating: sedimentation introduces viscous resistance to beating that is stronger in the up phase of the helicoidal cycle than in the down phase, resulting in velocity-dependent reorientation.

In addition to these hydrodynamic mechanisms, physiological mechanisms have been postulated. It has been observed that *Paramecium* swims slightly faster upwards than downwards, once sedimentation has been subtracted (Machemer et al., 1991; Ooya et al., 1992; Fig. 8*B*), and the avoiding reaction is triggered more often when it swims backwards than upwards, although this bias tends to disappear after some time (Nagel and Machemer, 2000; Fig. 8*C*). Although spurious correlations should be ruled out (e.g., cells that swim more slowly may tend to fall), Machemer and colleagues have proposed that this is because of pressure differences between the top and bottom ends of the cell, which are sensed by mechanoreceptors. As there is a spatial gradient of mechanosensitivity between the front and rear, the transduced current would be hyperpolarizing when the anterior end is upward (increased pressure on the rear end) and depolarizing when the anterior end is downward. In support of this hypothesis, a cell vertically immobilized between two horizontal electrodes can spontaneously turn upward or downward, and small membrane potential changes with the expected sign are observed, although with long latency (on the order of 20 s; Gebauer et al., 1999). These physiologically induced changes in mean velocity and avoiding reaction rate likely represent a small contribution to gravitaxis, compared with the reorientation of the cell (Roberts, 2010), but it is conceivable that reorientation itself occurs by physiological modulation of velocity within the helicoidal cycle (Mogami and Baba, 1998).

### How *Paramecium* turns

In the avoiding reaction, *Paramecium* swims backward (if the reaction is strong) then turns before it swims forward again. Backward swimming occurs because cilia reorient, with the power stroke oriented toward the anterior end instead of the posterior end, but how can *Paramecium* turn? Turning requires some inhomogeneity in the ciliary beating pattern.

**Figure 10.**
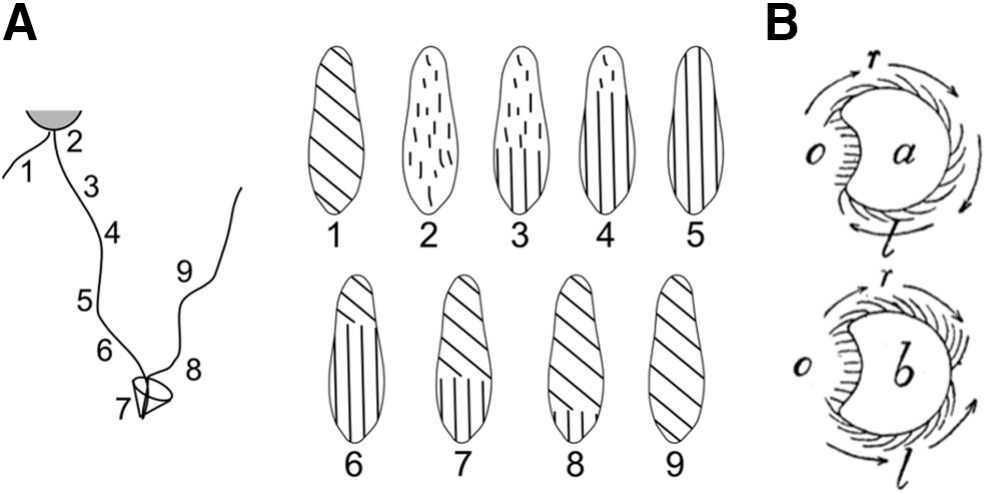
Details of the avoiding reaction. ***A***, Reorganization of the ciliary beating pattern during the avoiding reaction (after Machemer, 1969). ***B***, Cross-section of *Paramecium* seen from the anterior end, during forward swimming (***a***, corresponding to step 1) and during reorientation (***b***, corresponding to step 6), according to Jennings (1904). The arrows correspond to the induced movement of the body (opposite to the beating direction).

First, anterior and posterior cilia do not revert synchronously during the avoiding reaction (Fig. 10*A*; Párducz, 1967; Machemer, 1969). When the avoiding reaction is initiated, all cilia simultaneously strike forward, which moves the cell backward (2). The beating pattern then progressively reorganizes into the metachronal pattern as the cell swims backward (3–5). Reorientation of the cell starts when the anterior end reverts to the forward metachronal pattern (6–8). Thus, anterior and posterior ends show different metachronal patterns, respectively, of forward and backward swimming.

It is not obvious, however, how this asynchronous pattern would make the cell turn. If the beating pattern were axisymmetric, then the net force produced by either group of cilia (anterior or posterior) should be directed along the main axis. Jennings claims that cilia in the oral groove may also reverse, i.e., they expel fluid from the mouth (Jennings, 1899a; Fig. 8*C*). This could make *Paramecium* turn toward its aboral (dorsal) side, as observed, but Jennings and Jamieson observed that when *Paramecium* was cut in two pieces below the oral groove, both pieces could turn in a similar way (Jennings and Jamieson, 1902). Jennings also mentions that cilia of the anterior end do not all strike to the right: instead, they strike toward the oral groove (Jennings, 1904; Fig. 10*B*). As a result, the cell turns toward the aboral side. This is supported by more recent observations in a flattened ciliary sheet from *Paramecium* (Noguchi et al., 1991). Thus, turning likely results from inhomogeneity in the response of different groups of cilia, but details are still lacking.

## The Physiologic Basis of Behavior

### The action potential

When *Paramecium* touches an obstacle, mechanosensitive channels open, depolarize the membrane and trigger a calcium-based action potential (Eckert, 1972). The entry of calcium then triggers the reorientation of cilia, so that *Paramecium* swims backwards. Then calcium is buffered or pumped out (Plattner et al., 2006; Yano et al., 2015) and the cilia reorient in the original direction.

Historically, *Paramecium* electrophysiology has been studied by placing the cell in a tiny droplet, letting the fluid evaporate until the cell is captured by surface tension, then inserting sharp microelectrodes and covering with extracellular medium (Naitoh and Eckert, 1972). A recent method immobilizes the cell by suction against a filter (Kulkarni et al., 2020).

*Paramecium* is an isopotential cell, as demonstrated with two-electrode measurements (Eckert and Naitoh, 1970; Dunlap, 1977; Satow and Kung, 1979), which is a particularly favorable situation for electrophysiological modeling. This can be sensed from an estimation of the electrotonic length $\lambda =\sqrt{\frac{dr_{m}}{4R_{i}}}$, where *d* is diameter, *r_m_* is specific membrane resistance, and *R_i_* is intracellular resistivity. For *P. tetraurelia*, cell width is 34 μm (Nagel and Machemer, 2000), with r_m_ = 64,000 Ω. cm^2^ (Dunlap, 1977) and *R_i_* = 500 Ω. cm (conservative estimate based on the ∼5 lower intracellular ionic content compared with mammals), we obtain λ≈ 330 mm, much larger than the cell’s length (115 μm). In the same way, for a 200 nm wide cilium, we obtain λ≈ 260 μm, much larger than its 10-μm length.

*Paramecium* has a resting potential of about −30 to −20 mV (more depolarized than neurons), depending on the extracellular medium (Naitoh and Eckert, 1968a). *P. caudatum* has a capacitance of ∼700 pF, half of which is due to the cilia (Machemer and Ogura, 1979), and a resistance of ∼65 M*Ω* (again depending on the extracellular medium), giving a membrane time constant of ∼45 ms. *P. tetraurelia*, which is smaller, has a resistance of ∼45–60 M*Ω* (Satow and Kung, 1976; Nagel and Machemer, 2000). Capacitance is not documented, but a simple scaling based on membrane area (Machemer and Ogura, 1979; Nagel and Machemer, 2000) gives ∼300 pF. These values are consistent with the surfacic capacitance of other cells including neurons (∼1 μF/cm^2^).

The negative resting potential is due to a high intracellular concentration of K^+^ ions (18–34 mm depending on studies; Naitoh and Eckert, 1969, 1973; Hansma, 1974; Oertel et al., 1978; Ogura and Machemer, 1980; Oka et al., 1986), much larger than the extracellular concentration (typically ∼1–4 mm KCl in experiments; Machemer and Ogura, 1979; Machemer, 1998). Conversely, there is a low intracellular concentration of Ca^2+^ ions at rest (50–200 nm; Klauke and Plattner, 1997; Iwadate, 2003), while the extracellular concentration is orders of magnitude higher (the minimal viable concentration is ∼0.1 mm; Naitoh and Eckert, 1968a). At rest, the membrane is permeable to many cations (Naitoh and Eckert, 1968a). Thus, the ionic content of the cytosol is approximately five times lower than metazoan cells (where intracellular K^+^ concentration is ∼150 mm). One reason might be that the extracellular medium (fresh water) typically has very low ionic content, so that the cytosolic ions exert a large osmotic pressure on the membrane. In *Paramecium* and other protozoa, this osmotic imbalance is regulated by specialized organelles, the contractile vacuoles, which expel water that invades the cell by osmosis (Allen and Naitoh, 2002).

**Figure 11.**
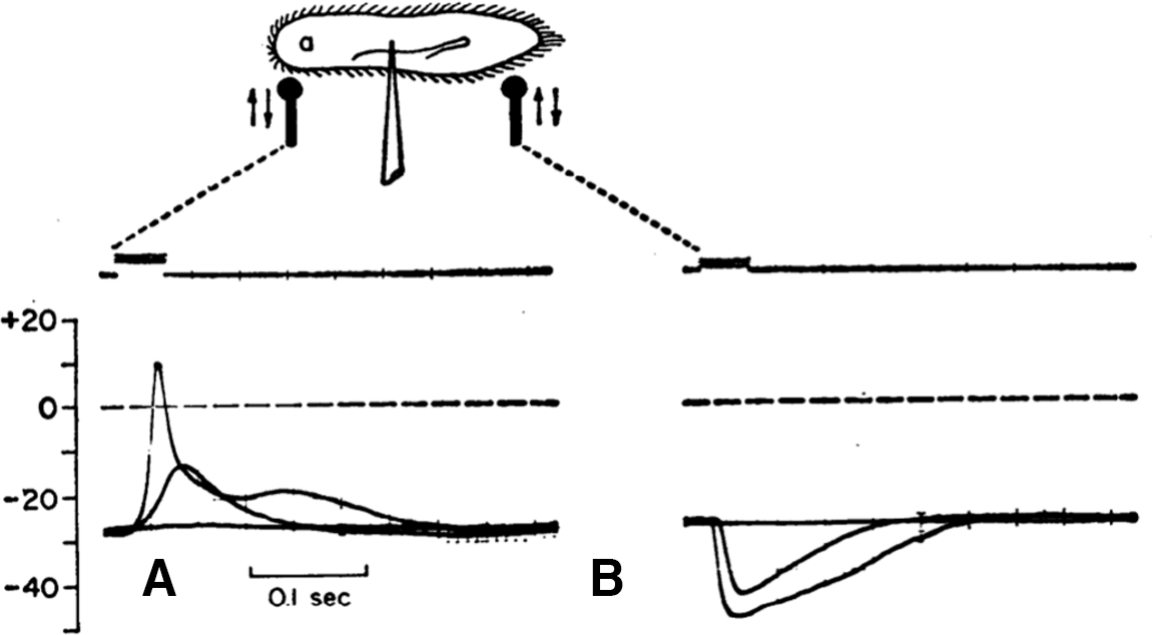
Membrane potential responses to mechanical stimulation with a glass stylus on the front (***A***) and on the rear (***B***; from Naitoh and Eckert, 1969, with permission; top traces: voltage command to the piezoelectric actuator).

**Figure 12.**
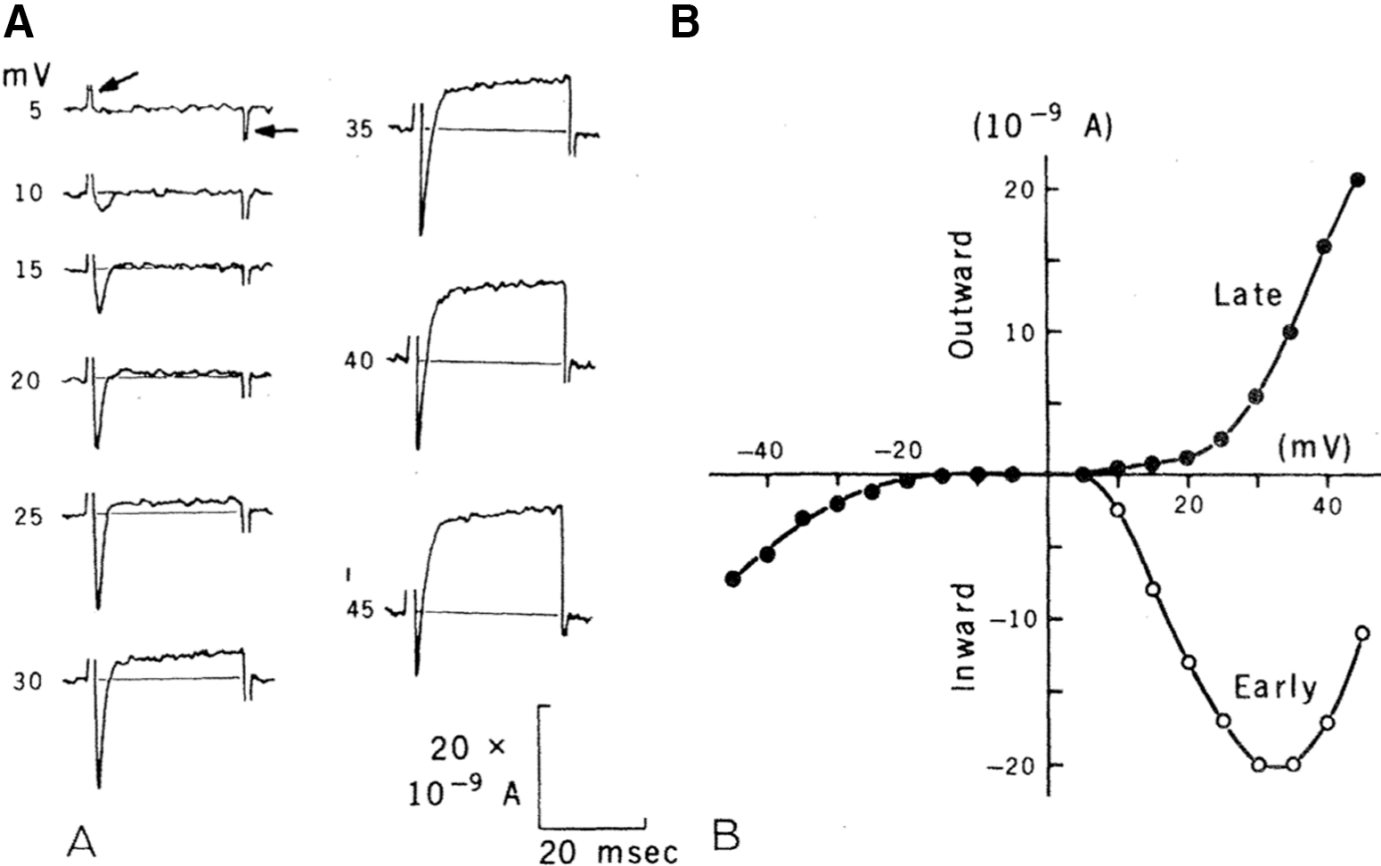
Action potential currents in *P. caudatum* (from Brehm and Eckert, 1978a, with permission). ***A***, Current recorded in voltage-clamp with different depolarization steps above resting potential. The first and last peaks are capacitive transients. The early negative transient is mediated by calcium; the late positive current is mediated by potassium. ***B***, Early and late currents versus membrane potential (relative to rest).

When *Paramecium* is mechanically stimulated on the front, or a current is injected, the membrane is depolarized (Fig. 11). If the stimulus is strong enough, this depolarization triggers a graded action potential, with a stimulus-dependent amplitude (all-or-none spikes can occur if extracellular calcium is partially replaced by barium; Naitoh and Eckert, 1968b). This action potential is due to calcium voltage-gated channels distributed over the cilia and delayed rectifier potassium channels located in the somatic membrane; this can be demonstrated by removing the cilia with ethanol and shaking (Machemer and Ogura, 1979). In response to a voltage step, the cell produces a current consisting of two phases: a fast inward current carried by Ca^2+^, and a slower outward current carried by K^+^ (Fig. 12), which have been separated using behavioral mutants (Saimi and Kung, 1987).

The Ca^2+^ current inactivates quickly (a few milliseconds) by a calcium-dependent mechanism: the entry of calcium (rather than voltage) inactivates the channels (Eckert and Brehm, 1979; Brehm et al., 1980; Eckert and Chad, 1984), there is also a slower voltage-gated inactivation acting over tens of seconds (Hennessey and Kung, 1985). Recovery from inactivation takes a few tens to a hundred of milliseconds (Naitoh et al., 1972; Brehm et al., 1980). This is a common form of inactivation of calcium channels in neurons, which has been discovered first in *Paramecium* (Brehm and Eckert, 1978a). It involves calmodulin, a highly conserved calcium sensor that is found across all species (Ben-Johny and Yue, 2014). Genetically, three related α units have been identified in the cilia (Lodh et al., 2016), which are similar to the Ca_V_1 mammalian family (L-type).

The voltage-gated K^+^ current is a delayed rectifier current, which activates quickly (a few milliseconds; Eckert and Brehm, 1979) and inactivates slowly (a few seconds; Satow and Kung, 1980; Saimi et al., 1983). There is also a calcium-activated current, which develops more slowly (Satow and Kung, 1980). It is involved in repolarization after sustained stimulation (Saimi et al., 1983). Genetic analysis has identified in particular SK channels located in the cilia (Valentine et al., 2012; Yano et al., 2013). All these channels have homologs in mammalian neurons.

Currents selective for Na^+^ (Saimi, 1986; Saimi and Ling, 1990) and Mg^2+^ (Preston, 1990, 1998) have also been identified.

### Electromotor coupling

Cilia are highly conserved structures. Motile cilia are found not only in swimming microorganisms but also in multicellular organisms including humans, where they are involved in moving fluids, for example the cerebrospinal fluid (Faubel et al., 2016). The cilium contains a cytoskeleton called the axoneme, composed of nine microtubule doublets arranged in a ring around a central pair of microtubules (Porter and Sale, 2000). Dynein motors make microtubule doublets slide on each other, which bends the cilium (Walczak and Nelson, 1994). The activity of these motors is regulated by second messengers, in particular calcium and cyclic nucleotides (cAMP and cGMP).

**Figure 13.**
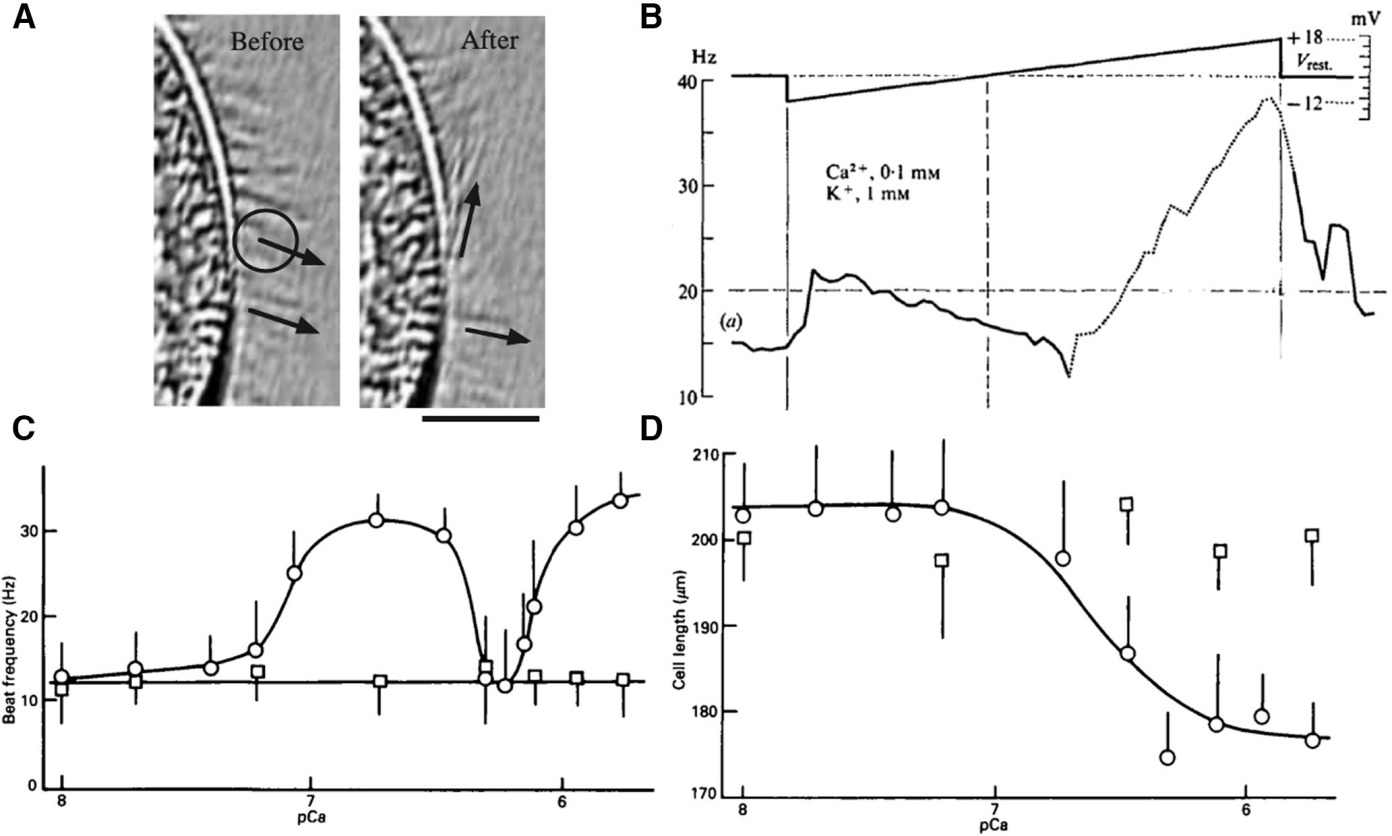
Electromotor coupling. ***A***, Calcium uncaging in cilia (circle) triggers local ciliary reversal (from Iwadate, 2003, with permission). ***B***, Beating frequency (filled: positive; open: negative) as a function of membrane potential in voltage clamp (from Machemer, 1976, with permission). Reversal is indicated by dots. ***C***, Beating frequency versus pCa (-log_10_ [Ca^2+^]) in a permeabilized cell (from Nakaoka et al., 1984, with permission). Squares and circles are two different permeabilized models, circles being more physiological. Cilia reverse at the minimum beating frequency. ***D***, Cell length versus pCa in a permeabilized cell (from Nakaoka et al., 1984, with permission).

In the absence of stimulation, cilia beat at ∼10–20 Hz with a power stroke toward the right and rear of the cell. Ciliary reversal is triggered by calcium entering the cell through voltage-gated calcium channels distributed over the cilia: this has been shown by direct intracellular exposure of cilia to [Ca]_i_ > 1 μm (Naitoh and Kaneko, 1972; by making the membrane permeable with a detergent) and calcium uncaging in the cilia (Iwadate, 2003; Fig. 13*A*). In *P. tetraurelia*, Oertel et al. (1977) estimated that the largest calcium current triggered by a short voltage step increases the ciliary calcium concentration by ∼20 μm, which then decays because of buffering and pumping. Thus, stronger current pulses trigger larger and faster spikes, resulting in larger calcium increase and therefore longer reversed beating (Machemer and Eckert, 1973). Cyclic nucleotides (cAMP and cGMP) antagonize ciliary reorientation, i.e., an increased cAMP concentration raises the voltage threshold for ciliary reorientation (Nakaoka and Machemer, 1990).

Beating frequency also changes with voltage (Machemer and Eckert, 1975; Fig. 13*B*). In particular, cilia beat faster when the command voltage is increased above resting potential. Early work in permeabilized cells indicated that calcium controls ciliary reorientation but not beating frequency (Naitoh and Kaneko, 1972), but this was later argued to be because of unphysiological aspects of the permeable models (Nakaoka et al., 1984). In more physiological permeabilized cells, an increase in ciliary calcium concentration above the resting level triggers ciliary reorientation and an increase in beating frequency, matching the effect of depolarization (Fig. 13*C*). Note that swimming velocity does not exactly follow this frequency increase, because it also depends on the coordination of cilia, which is disrupted when cilia reorient. For small depolarizations, not all cilia reorient (Machemer and Eckert, 1975), which may explain how the organism turns. The cell also contracts when calcium concentration increases (Fig. 13*D*).

### Mechanotransduction

Mechanoreception in *Paramecium* and other ciliates has been the object of several reviews (Naitoh, 1984; Machemer, 1985; Machemer and Deitmer, 1985; Deitmer, 1992). Touching the anterior part of *Paramecium* results in membrane depolarization, while touching the posterior part results in membrane hyperpolarization (Naitoh and Eckert, 1969). Six genes of the Piezo family (Coste et al., 2010) have been identified in the genome, similar to those mediating mechanosensitivity in many species including mammals. Ionic channels mediating mechanosensitivity are located on the basal membrane; a deciliated cell is still mechanosensitive (Ogura and Machemer, 1980). Cilia are not directly involved in transduction, but they are involved in the mechanical transfer and filtering of stimuli. For example, the tail has long immobile cilia, which may enhance mechanical sensitivity (in particular to current flows) by spreading the mechanical stimulation over a larger membrane area (Machemer and Machemer-Röhnisch, 1984; Machemer-Röhnisch and Machemer, 1984).

**Figure 14.**
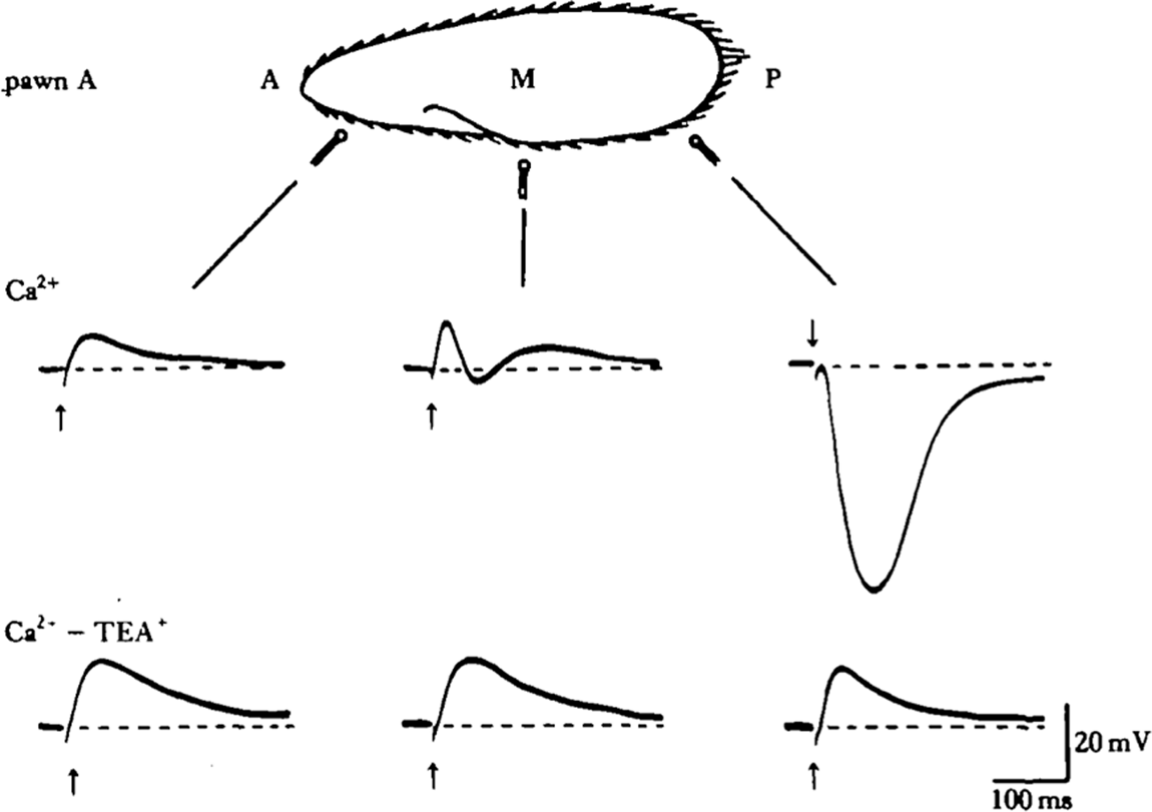
Mechanosensitive responses measured as a function of stimulation position (A: anterior; P: posterior) in a *P. aurelia* mutant with no action potential (from Satow et al., 1983, with permission). Below, K^+^ currents are blocked with TEA.

Mechanosensitive currents change gradually from the posterior to anterior part because of overlapping spatial gradients of K^+^ and Ca^2+^ mechanosensitive channels (Ogura and Machemer, 1980; Satow et al., 1983). Currents in the posterior part are mostly carried by fast K^+^ currents (time constant in the 10-ms range), whereas currents in the anterior part are carried mainly by Ca^2+^ (and possibly other divalent cations; Satow et al., 1983) and are slower (in the 20-ms range; Fig. 14). In the middle region, a mixed current can be observed, with an outward then inward component, indicative of a superposition of two ionic channel responses. There are also graded changes in mechanosensitivity along the oral-aboral (dorsoventral) axis.

Mechanical responses have been studied mainly by deflecting a thin glass stylus onto the membrane with a piezo-electric actuator. In another ciliate, *Stylonichia*, the transduced current increases linearly with the deflection amplitude of the probe; the resulting potential may saturate for strong stimuli, near the reversal potential. Faster deflections reduce response latency without changing the amplitude. When a mutant with defective ciliary calcium channels is mechanically stimulated, ciliary reversal is observed only at the site of stimulation on the anterior membrane (Takahashi and Naitoh, 1978): this indicates that mechanical stimulation only recruits local mechanoreceptors (these can trigger ciliary reversal because the transduced current is carried by calcium). Stimulations integrate both spatially and temporally, with no sign of refractoriness. Finally, the duration of the deflection has no effect on the response. The termination of the current may be due to an adaptation process and/or to the membrane passively retreating from the probe.

Thus, the integration of mechanical stimuli is analog to synaptic integration in a neuron: stimulation at a site produces a transient current through ionic channels, transduced currents are integrated both spatially and temporally, and the resulting potential response may trigger an action potential if it is large enough.

### Electrophysiology of the escape reaction

When *Paramecium* is mechanically stimulated on the rear, the membrane is hyperpolarized (Fig. 14), which then triggers the escape reaction: swimming velocity increases. The electrophysiological response is shaped by several hyperpolarization-activated channels.

A fast inward rectifier K^+^ current is activated by hyperpolarization, most strongly below E_K_ (Oertel et al., 1978), and partially inactivates over a few hundred milliseconds (Preston et al., 1990). Thus, the activation voltage depends on extracellular K^+^ concentration. If that concentration is very low, a regenerative hyperpolarization can be obtained (Satow and Kung, 1977). This current is similar to inward rectifiers found in other species (Doupnik et al., 1995). Another K^+^ current activates with calcium (Preston et al., 1990).

A calcium current activates with hyperpolarization, and the entry of calcium then mediates an increase in beating frequency (Nakaoka and Iwatsuki, 1992; Preston et al., 1992a,b). This current actually activates within a few tens of ms, and decays more slowly through calcium-dependent inactivation (Preston et al., 1992b). It actually consists of two pharmacologically distinct currents located in the somatic membrane, one of which is sustained (Nakaoka and Iwatsuki, 1992). The magnitude of the hyperpolarization-activated calcium current is directly related to the increase in beating frequency, and blocking this current also blocks the frequency increase (Nakaoka and Iwatsuki, 1992). Thus, it appears that beating frequency is controlled by calcium concentration in the somatic membrane, presumably at the base of cilia, in line with studies in other ciliary systems (Tamm, 1994). This contradicts several earlier hypotheses: that beating frequency increases with a hyperpolarization-induced decrease in ciliary calcium concentration (Machemer, 1974), by a iontophoretic mechanism in the cilia (Brehm and Eckert, 1978b), or by regulation by cyclic nucleotides (Satir et al., 1993; Pech, 1995). The latter hypothesis did receive some support (Bonini et al., 1986; Hamasaki et al., 1991; Schultz et al., 1992), as raising cAMP concentration makes cilia beat faster, but it has been disproven by the demonstration that, when the cell’s voltage is maintained constant, injecting high levels of cAMP has no effect on beating frequency (Hennessey et al., 1985; Nakaoka and Machemer, 1990). Thus, the effect of cAMP was likely indirectly due to the hyperpolarization induced by cAMP (Bonini et al., 1986).

## Discussion

Gomez-Marin and Ghazanfar described three fundamental biological principles of behavior that highlight the need for integrated approaches in neuroscience: materiality, agency and historicity (Gomez-Marin and Ghazanfar, 2019). Materiality refers to the role of body and environment in behavior. That is, the relation between neural activity and behavior is not just a case of correspondence (the coding view; Brette, 2019), but also of physical causality: spikes cause particular physiological effects, the results of which are determined by the structure of the body and the environment it interacts with (Tytell et al., 2011). For example, in *Paramecium*, cilia are under electrical control but efficient motor coordination is partly achieved by hydrodynamic interactions between cilia. Agency refers to the fact that action and perception form a closed loop in the service of goals, rather than a linear stimulus-reaction chain. For example, when *Paramecium* meets an obstacle, the mechanosensory signal is determined not just by the object but also by the motor response that the signal causes, in a closed loop. This concept is increasingly appreciated in cognitive science, philosophy of mind and more recently neuroscience (Maturana and Varela, 1973; Powers, 1973; Gibson, 1979; Brooks, 1991; Bickhard and Terveen, 1996; Hurley, 2001; O’Regan and Noë, 2001; Ahissar and Assa, 2016; Pezzulo and Cisek, 2016; Brette, 2019). Historicity refers to the fact that organisms are individuals: variability is best understood not as a noisy deviation around a norm but as a functional result of their history. In *Paramecium*, this is evident for example in long-term adaptation to new environments, but also in some exploratory behaviors (such as tube escape).

Addressing these three principles requires studying an entire organism in an environment, rather than isolated subsystems. Computational neuroethology is a subfield of computational neuroscience focusing on the modeling of autonomous behavior (Beer, 1990), which has been investigated in particular artificial organisms (Beer and Gallagher, 1992) and robots (Webb, 2001). More recently, integrated models of *C. elegans* (Izquierdo and Beer, 2016; Cohen and Denham, 2019), *Hydra* (Dupre and Yuste, 2017; Wang et al., 2020), and jellyfish *Aurelia aurita* (Pallasdies et al., 2019) have been developed. Those model organisms have certain obvious advantages over *Paramecium*, namely the fact that they have a nervous system, with interacting neurons. But *Paramecium* has great assets for integrative modeling of a whole organism, relating physiology and behavior.

First, there is an extensive literature on *Paramecium*, covering detailed aspects of behavior, genetics, electrophysiology, cell and molecular biology. This literature has highlighted similarities with metazoans, in particular nervous systems, not only functionally but also at genetic and molecular levels (Connolly and Kerkut, 1983; Hinrichsen and Schultz, 1988; Beisson et al., 2010b; Yano et al., 2015; Plattner and Verkhratsky, 2018), with similar ionic channels, pumps, signaling pathways (calcium, cyclic nucleotides), sensory receptors, even GABA receptors. Second, it benefits from various tools, for example genetic tools such as RNA interference (Galvani and Sperling, 2002), proteomics (Yano et al., 2013), and whole genome sequencing (Aury et al., 2006; Arnaiz et al., 2010; Arnaiz and Sperling, 2011; McGrath et al., 2014), behavioral monitoring (Drescher et al., 2009), immobilization for electrophysiology (Kulkarni et al., 2020). Finally, it is easy to culture (Beisson et al., 2010a), it has a rich behavior that can be easily observed and quantified, and it allows intracellular electrophysiology in an intact organism, while monitoring its behavior.

As outlined in this review, a number of neuroscientific themes can be addressed and revisited in *Paramecium.* One such theme is the physiological basis of behavior and the relation between perception and action. A classical way to frame this problem is what Susan Hurley called the “classical sandwich” (Hurley, 2001): at the periphery, a perceptual system transforms stimuli into representations and a motor system transforms motor representations into actions; sandwiched between perception and action, cognition manipulates representations. As noted by many authors, the classical sandwich has many conceptual issues (Gibson, 1979; Brooks, 1991; O’Regan and Noë, 2001; Pezzulo and Cisek, 2016; Brette, 2019). Cisek, for example, noted that it leaves the cartesian dualistic view essentially unchanged, replacing the non-physical mind by “cognition” while preserving problematic homuncular concepts (Cisek, 1999). Another key issue is that framing neural activity as responses to stimuli denies any autonomy to the organism. As Dewey pointed out (Dewey, 1896), sensory signals are as much causes as consequences of the organism’s activity, because the relation between organism and environment is one of coupling rather than command. By its relative simplicity, *Paramecium* offers the possibility to study the physiological basis of autonomous behavior outside the frame of the classical sandwich, because it seems feasible to develop closed-loop dynamical systems models of the organism behaving autonomously in an environment, where spikes are not symbols but actions (Brette, 2019).

Motor control is a related theme where *Paramecium* may provide some insights. Embodiment is the idea that the body can contribute to motor control, beyond the mere execution of central commands. In *Paramecium*, cilia beat in a coordinated fashion in the absence of central command, by hydrodynamic and mechanical interactions, yielding efficient swimming. More generally, the mechanical properties of its body contribute to its navigation abilities, as when navigating in confined spaces, and more generally when interacting with surfaces. As it turns out, *Paramecium* appears to use neither of the two mainstream concepts in motor control, planning (or feedforward control; Wolpert and Ghahramani, 2000) and feedback control (Powers, 1973). Instead, it uses another way to produce goal-directed behavior, based on the Darwinian insight that random exploration and elimination of unsuccessful attempts can produce adapted behavior. This simple principle allows *Paramecium* to perform non-trivial sensorimotor tasks with a single “neuron”.

While the physiological basis of learning is classically framed in terms of stimulus association, *Paramecium* may offer the possibility to address it in a more ecological context, that is, autonomous learning of a task. Tube escape might be such a task; however, the learning capabilities of *Paramecium* are still somewhat unclear.

As *Paramecium* is both an organism and a cell, it also offers the opportunity to investigate the relation between cellular plasticity and behavioral plasticity. Intrinsic plasticity is well documented in neurons (Daoudal and Debanne, 2003), but it remains very challenging to understand its functional implications for the organism. Thus, it is classically interpreted in terms of homeostasis of cellular properties (e.g., firing rate), or of abstract information-theoretical properties. In *Paramecium*, since the relation between cellular physiology and behavior is more direct than in brains, it becomes possible to relate intrinsic plasticity with behavioral plasticity. For example, ionic channel properties adapt to changes in temperature in such a way as to preserve normal motor behavior (Nakaoka et al., 1982; Martinac and Machemer, 1984). Similarly, developmental plasticity can be addressed by investigating the physiological and behavioral changes after fission (Iftode et al., 1989). Indeed, as ionic channels are spatially organized (for example depolarizing mechanoreceptors at the front), this organization is disrupted by fission and must be somehow restored.

This opens exciting perspectives for the development of integrated models of a “swimming neuron”.

## Synthesis

Reviewing Editor: Juan Burrone, King’s College London Decisions are customarily a result of the Reviewing Editor and the peer reviewers coming together and discussing their recommendations until a consensus is reached. When revisions are invited, a fact-based synthesis statement explaining their decision and outlining what is needed to prepare a revision will be listed below. The following reviewer(s) agreed to reveal their identity: Kirsty Wan.

This manuscript provides a detailed review of what can be considered an extreme version of a reductionist neuronal system - a single-cell organism. When searching for simpler systems to study physiology and behaivour, none can be simpler than the paramecium. And yet both the behaviour and physiology of this single-celled organism are also very rich. This work describes in detail the many behaviours of mainly two paramecia P. caudatum and P. aurelia. and links this behaviour to their physiology, which includes a number of voltage-gated channels that decode external inputs and convert it into motion. This extensive review is well written, thought provoking and should of interest to a wide scientific community. Below are some specific comments on the manuscript.

Neuroscience is often, almost by definition, predicated upon knowledge and insights gained from various animal models with well-studied nervous systems. The simplest organisms are often overlooked, yet are capable of surprisingly complex behaviours. The ethology of unicells had been the subject of intense study several decades ago, but is now experiencing something of a resurgence in interest.

This comprehensive review summarises and synthesises much of the data available in the literature about the bioelectrical basis of behaviour in the model ciliate Paramecium. The work will be of interest to a broad audience, particularly biophysicists working on cell motility, while serving to (re)acquaint neuroscientists with this unique model system. Some mechanisms are vague or only hypothetical at this stage, but this is understandable given lack of data.

I have some recommendations that could improve the paper.

1. The work is very much a synthesis of old results - all figures are taken directly from the old literature. I would have liked to see a more elaborate discussion of what this could mean from the ‘integrative neuroscience’ perspective. How might the (motor) behaviour of paramecium be interpreted from the perspective/language of neuronal control principles? There is some mention of this in the last section, but this can be expanded, especially for the intended neuroscience readership.

2. The entire paper focuses on Paramecium, while this may be justified, it would be useful to highlight any conserved features or comparisons with other systems (in the discussions but also throughout) - this may include other ciliated organisms, or other model organisms typically featured in neuroscience. For instance, details of the capacitance of the cell are given (3.1), but how do these values compare with other cell types - ‘true’ neurons? What about the nature, identity, directionality of ionic currents, resting state of membranes etc, how do all these compare with ‘true’ neurons? Which receptors are shared by paramecium and metazoan neuron-types?

3. ‘many signalling pathways of neurons have been found in Paramecium’ - can these connection be expanded? again with above comments in in mind.

4. section 1 - characterisation of the swimming mechanism/trajectories seem to be based on old literature from Jennings et al, is there anything more recent? particularly, is it certain that the oral grooves always faces the inside of the spirals? what is known about the handedness - do all paramecia have the same handedness - is it fixed or can the same individual switch, for example when reversing or responding to cues?

5. Again, many descriptions originate from stylised accounts by Jennings, it would be good to check if these observations indeed hold true, e.g. provide more recent references, if any. Fig 4 - is there other evidence of this type of graded response? what happens when organisms interact with boundaries - where does that fit into the classification? Similarly does turning always happen on the same side?

6. Ciliates often contract - which provides another route for turning/other reactions, this isn’t really mentioned - a contractile cytoskeleton may also respond to membrane potential? is there any evidence of this?

7. The authors may wish to revisit and clarify what they mean by task-driven “trial and error”. After all, bacterial chemotaxis can also be considered a form of trial and error. What is different here (1.4)? If repeated encounters with an obstacle leads to a ‘reversal’, then it can just repeat this until *by chance* it manages to escape. So the question really is whether these patterns of reversal, or orientations say, actually change over successive trials?

minor points

Some sentences/concepts are repeated - the authors are advised to proofread to remove any redundancies.

are calcium channels located exclusively in the cilia?

the authors speak of a dorsal-ventral axis, is this the correct term for unicellular organisms, what about oral-aboral?

does ejection of trichocysts really lead to propulsion? (section 1.2)

It is stated that the cell should be isopotential, a few explanatory sentences may be useful to explain why this should necessarily be the case.

fig 11, in BW it’s very hard to tell the [Ca2+] and [K+] curves apart.

On page 7, instead of ‘releases GABAB’ it should read ‘releases GABA’

## Author Response

Response to reviewers

Dear editor and reviewers,

Thank you for these very relevant remarks. In this revision, I have expanded the discussion of the bridges between Paramecium neuroscience and mainstream neuroscience. Regarding the behavior, much of what is known is indeed very old and in need of more modern studies. I have tried to emphasize this fact. I have also responded to each remark in detail. Unrelated to these remarks, I redrew Fig. 10A for copyright reasons.

I hope you will like this revised version.

Best regards,

Romain Brette

Detailed response

This manuscript provides a detailed review of what can be considered an extreme version of a reductionist neuronal system - a single-cell organism. When searching for simpler systems to study physiology and behaivour, none can be simpler than the paramecium. And yet both the behaviour and physiology of this single-celled organism are also very rich. This work describes in detail the many behaviours of mainly two paramecia P. caudatum and P. aurelia. and links this behaviour to their physiology, which includes a number of voltage-gated channels that decode external inputs and convert it into motion. This extensive review is well written, thought provoking and should of interest to a wide scientific community. Below are some specific comments on the manuscript.

Neuroscience is often, almost by definition, predicated upon knowledge and insights gained from various animal models with well-studied nervous systems. The simplest organisms are often overlooked, yet are capable of surprisingly complex behaviours. The ethology of unicells had been the subject of intense study several decades ago, but is now experiencing something of a resurgence in interest.

This comprehensive review summarises and synthesises much of the data available in the literature about the bioelectrical basis of behaviour in the model ciliate Paramecium. The work will be of interest to a broad audience, particularly biophysicists working on cell motility, while serving to (re)acquaint neuroscientists with this unique model system. Some mechanisms are vague or only hypothetical at this stage, but this is understandable given lack of data.

I have some recommendations that could improve the paper.

1. The work is very much a synthesis of old results - all figures are taken directly from the old literature. I would have liked to see a more elaborate discussion of what this could mean from the ‘integrative neuroscience’ perspective. How might the (motor) behaviour of paramecium be interpreted from the perspective/language of neuronal control principles? There is some mention of this in the last section, but this can be expanded, especially for the intended neuroscience readership.

Regarding motor control, there are two paragraphs in section 1.4, which I have now expanded a little. I have also expanded the discussion to address the broader issue.

2. The entire paper focuses on Paramecium, while this may be justified, it would be useful to highlight any conserved features or comparisons with other systems (in the discussions but also throughout) - this may include other ciliated organisms, or other model organisms typically featured in neuroscience. For instance, details of the capacitance of the cell are given (3.1), but how do these values compare with other cell types - ‘true’ neurons? What about the nature, identity, directionality of ionic currents, resting state of membranes etc, how do all these compare with ‘true’ neurons? Which receptors are shared by paramecium and metazoan neuron-types?

I have added some comparisons throughout the paper. There is work on other ciliates, for example Stentor, but I do not know it as well. Generally, electrophysiology is much more developed in Paramecium than in other ciliates, but there is very interesting behavioral work on various ciliates.

3. ‘many signalling pathways of neurons have been found in Paramecium’ - can these connection be expanded? again with above comments in in mind.

I have added some detail.

4. section 1 - characterisation of the swimming mechanism/trajectories seem to be based on old literature from Jennings et al, is there anything more recent? particularly, is it certain that the oral grooves always faces the inside of the spirals? what is known about the handedness - do all paramecia have the same handedness - is it fixed or can the same individual switch, for example when reversing or responding to cues?

Indeed, most behavioral work has been done by Jennings and contemporary scientists (note however that there are a number of more recent references in this section). There are a number of 20th century papers showing trajectories, but not so much their fine structure. Specifically, the observation that the oral groove faces the spiral axis has been reported after Jennings by a couple of authors, in particular by Bullington (ref. 153), but this is also quite old (1930) and to my knowledge it has not been quantified. This is clearly an area where further work would be useful, in particular with high-quality 3D recordings. Regarding the handedness, as far as I know it is always the same, but again this has not been quantified systematically. I added some comments about this issue.

5. Again, many descriptions originate from stylised accounts by Jennings, it would be good to check if these observations indeed hold true, e.g. provide more recent references, if any. Fig 4 - is there other evidence of this type of graded response? what happens when organisms interact with boundaries - where does that fit into the classification? Similarly does turning always happen on the same side?

Regarding graded responses, there is evidence about the gradedness of backward swimming in the electrophysiological literature, that is, in observations of ciliary reversal in immobilized cells. I have added a reference (which was previously only mentioned in section 3). It is easy to observe in swimming cells, but to my knowledge it has not been quantified systematically. About turning: Jennings is categorical in his writings that it always happens on the same side, but to my knowledge this also remains to be measured systematically. In brief, there are gaps in the literature regarding detailed aspects of behavior (there is much more detailed knowledge about electrophysiology).

Regarding boundaries (I assume the reviewer means mechanical boundaries), as I wrote at the beginning of the section, Paramecium often gives the avoiding reaction when it hits an obstacle. However, it is not clear how this encounter relates to the intensity of the reaction. Then in addition to this active response, there are also hydromechanical interactions. Clearly sometimes paramecia slide along an object without giving the avoiding reaction (our observations). There is some literature on these effects in other ciliates, and in the physics literature.

I have added comments and a few references in the revised text.

6. Ciliates often contract - which provides another route for turning/other reactions, this isn’t really mentioned - a contractile cytoskeleton may also respond to membrane potential? is there any evidence of this?

Absolutely. This is very briefly mentioned at the end of section 3.2 and in 1.2 (“This speed increase is accompanied by a contraction along the longitudinal axis (52)”). To my knowledge, it has only been reported in electrophysiological studies of immobilized cells. This is presumably because it happens during the escape reaction, and therefore is probably very difficult to notice in motile cells.

7. The authors may wish to revisit and clarify what they mean by task-driven “trial and error”. After all, bacterial chemotaxis can also be considered a form of trial and error. What is different here (1.4)? If repeated encounters with an obstacle leads to a ‘reversal’, then it can just repeat this until *by chance* it manages to escape. So the question really is whether these patterns of reversal, or orientations say, actually change over successive trials?

Yes, run-and-tumble behavior of bacteria shares some similarity with Paramecium chemotaxis, except that Paramecium withdraws before changing direction, and has graded reactions. Berg (1975) describes the difference in the following way: “It was thought for many years that bacteria back up or choose new directions at random on entering regions which are unfavourable, that is, that the motor reflex is an avoidance reaction. This has not proved to be the case for E. coli. [...] As the concentration increases, the bacteria change direction less frequently; as it decreases, they swim as they do in the absence of a stimulus”.

In particular, it appears that Paramecium’s behavior is more deterministic than the behavior of bacteria. Biophysically, the membrane area is roughly 2 orders of magnitude larger in Paramecium than in E. Coli, so we expect that signal-to-noise ratio is about one order of magnitude larger. This certainly makes a difference in terms of sensing. Schematically speaking, bacterial run-and-tumble might be described as biased randomness (modulation of the rate of tumbling) whereas Paramecium behavior is perhaps better described as exploration and decision.

Another difference, which is maybe more a difference of degree than nature, is that the avoiding reaction is used in a variety of tasks; avoiding obstacles, thermal homeostasis, avoiding chemicals, etc. I am not sure bacterial behavior has the same diversity (but I am not an expert).

Here “trial-and-error” is just meant as: it tries a swimming direction, and if this leads to a bad or suboptimal situation, then it goes back and tries a different one. I assume the last question refers to learning, i.e., whether the “error” leads to a more persistent change in behavior. This is the aim of section 1.6. The situation described by the reviewer is closest to the behavior described briefly in section 1.2: when trapped in a narrow capillary with a dead end, Paramecium first does a few avoiding reactions, then after a minute, it suddenly starts making much stronger reactions (longer backward swimming).

My reading of Jennings is that to explain the behavior of Paramecium, one may call on the Darwinian insight that the selection of random “explorations” can lead to apparently goal-directed behavior, which at first sight one might be tempted to explain by planning (“intelligent design”) or by steering (Lamarckism) - somewhat analogs of feedforward and feedback control in motor neuroscience. Jennings speculates that the selection of actions is somehow stabilized physiologically, but I don’t think this has been established (we may speculate that this is what happens in the tube escape behavior).

I have expanded this section to address these issues.

minor points

Some sentences/concepts are repeated - the authors are advised to proofread to remove any redundancies.

Thank you, I have tried to eliminate these redundancies.

are calcium channels located exclusively in the cilia?

As far as I know, the calcium channels responsible for the avoiding reaction are only (or at least essentially) in the ciliary membrane, but there are other calcium channels in the somatic membrane (e.g. those described in section 3.4).

the authors speak of a dorsal-ventral axis, is this the correct term for unicellular organisms, what about oral-aboral?

Indeed, Jennings uses the term aboral. I thought dorsal and ventral might be more intuitive for many readers. I changed to oral/aboral, with dorsal/ventral in brackets.

does ejection of trichocysts really lead to propulsion? (section 1.2)

Yes, at a speed of 1-10 mm/s according to Hamel et al. In that paper (ref. 52), they explain the physics of the phenomenon.

It is stated that the cell should be isopotential, a few explanatory sentences may be useful to explain why this should necessarily be the case.

I added a calculation of electrotonic length.

fig 11, in BW it’s very hard to tell the [Ca2+] and [K+] curves apart.

That’s a misunderstanding, actually the legends for [Ca2+] and [K+] do not refer to two curves, but describe the composition of the extracellular medium (1 mM CaCl2 and 1 mM KCl). I removed it as it is confusing, and clarified the caption.

On page 7, instead of ‘releases GABAB’ it should read ‘releases GABA’

Indeed, thank you.
